# The impact of targeted malaria elimination with mass drug administrations on falciparum malaria in Southeast Asia: A cluster randomised trial

**DOI:** 10.1371/journal.pmed.1002745

**Published:** 2019-02-15

**Authors:** Lorenz von Seidlein, Thomas J. Peto, Jordi Landier, Thuy-Nhien Nguyen, Rupam Tripura, Koukeo Phommasone, Tiengkham Pongvongsa, Khin Maung Lwin, Lilly Keereecharoen, Ladda Kajeechiwa, May Myo Thwin, Daniel M. Parker, Jacher Wiladphaingern, Suphak Nosten, Stephane Proux, Vincent Corbel, Nguyen Tuong-Vy, Truong Le Phuc-Nhi, Do Hung Son, Pham Nguyen Huong-Thu, Nguyen Thi Kim Tuyen, Nguyen Thanh Tien, Le Thanh Dong, Dao Van Hue, Huynh Hong Quang, Chea Nguon, Chan Davoeung, Huy Rekol, Bipin Adhikari, Gisela Henriques, Panom Phongmany, Preyanan Suangkanarat, Atthanee Jeeyapant, Benchawan Vihokhern, Rob W. van der Pluijm, Yoel Lubell, Lisa J. White, Ricardo Aguas, Cholrawee Promnarate, Pasathorn Sirithiranont, Benoit Malleret, Laurent Rénia, Carl Onsjö, Xin Hui Chan, Jeremy Chalk, Olivo Miotto, Krittaya Patumrat, Kesinee Chotivanich, Borimas Hanboonkunupakarn, Podjanee Jittmala, Nils Kaehler, Phaik Yeong Cheah, Christopher Pell, Mehul Dhorda, Mallika Imwong, Georges Snounou, Mavuto Mukaka, Pimnara Peerawaranun, Sue J. Lee, Julie A. Simpson, Sasithon Pukrittayakamee, Pratap Singhasivanon, Martin P. Grobusch, Frank Cobelens, Frank Smithuis, Paul N. Newton, Guy E. Thwaites, Nicholas P. J. Day, Mayfong Mayxay, Tran Tinh Hien, Francois H. Nosten, Arjen M. Dondorp, Nicholas J. White

**Affiliations:** 1 Mahidol Oxford Tropical Medicine Research Unit, Faculty of Tropical Medicine, Mahidol University, Bangkok, Thailand; 2 Centre for Tropical Medicine and Global Health, Nuffield Department of Medicine, University of Oxford, Oxford, United Kingdom; 3 Shoklo Malaria Research Unit, Mahidol Oxford Tropical Medicine Research Unit, Faculty of Tropical Medicine, Mahidol University, Mae Sot, Thailand; 4 Institut de Recherche pour le Développement, Aix–Marseille University, INSERM, SESSTIM, Marseille, France; 5 Oxford University Clinical Research Unit, Wellcome Trust Major Overseas Programmes, Ho Chi Minh City, Vietnam; 6 Center of Tropical Medicine and Travel Medicine, Department of Infectious Diseases, Amsterdam University Medical Center, University of Amsterdam, Amsterdam, The Netherlands; 7 Lao–Oxford–Mahosot Hospital–Wellcome Trust Research Unit, Microbiology Laboratory, Mahosot Hospital, Vientiane, Lao People’s Democratic Republic; 8 Amsterdam Institute for Global Health & Development, Amsterdam, The Netherlands; 9 Savannakhet Provincial Health Department, Savannakhet Province, Lao People’s Democratic Republic; 10 Department of Clinical Tropical Medicine, Faculty of Tropical Medicine, Mahidol University, Bangkok, Thailand; 11 Department of Population Health and Disease Prevention, University of California, Irvine, Irvine, California, United States of America; 12 Maladies Infectieuses et Vecteurs: Écologie, Génétique, Evolution et Contrôle, Institut de Recherche pour le Développement, Université Montpellier, Montpellier, France; 13 Institute of Malariology, Parasitology, and Entomology, Ho Chi Minh City, Vietnam; 14 Center for Malariology, Parasitology and Entomology, Ninh Thuan Province, Vietnam; 15 Institute of Malariology, Parasitology, and Entomology, Quy Nhon, Vietnam; 16 National Center for Parasitology, Entomology and Malaria Control, Phnom Penh, Cambodia; 17 Provincial Health Department, Battambang, Cambodia; 18 Department of Pathogen Molecular Biology, London School of Hygiene & Tropical Medicine, London, United Kingdom; 19 WWARN Asia Regional Centre, Mahidol University, Bangkok, Thailand; 20 Department of Microbiology & Immunology, Yong Loo Lin School of Medicine, National University of Singapore, Singapore; 21 Singapore Immunology Network, Agency for Science, Technology and Research, Singapore; 22 Faculty of Medicine and Health Sciences, Linköping University, Linköping, Linköping, Sweden; 23 Wellcome Trust Sanger Institute, Hinxton, United Kingdom; 24 Department of Molecular Tropical Medicine and Genetics, Faculty of Tropical Medicine, Mahidol University, Bangkok, Thailand; 25 Department of Tropical Hygiene, Faculty of Tropical Medicine, Mahidol University, Bangkok, Thailand; 26 CEA–Université Paris Sud 11–INSERM U1184, IDMIT, Direction de la Recherche Fondamentale, Commissariat à l’Énergie Atomique et aux Énergies Alternatives, Fontenay-aux-Roses, France; 27 Centre for Epidemiology and Biostatistics, Melbourne School of Population and Global Health, University of Melbourne, Melbourne, Victoria, Australia; 28 Royal Society of Thailand, Bangkok, Thailand; 29 Myanmar Oxford Clinical Research Unit, Yangon, Myanmar; 30 Institute of Research and Education Development, University of Health Sciences, Vientiane, Lao People’s Democratic Republic; Burnet Institute, AUSTRALIA

## Abstract

**Background:**

The emergence and spread of multidrug-resistant *Plasmodium falciparum* in the Greater Mekong Subregion (GMS) threatens global malaria elimination efforts. Mass drug administration (MDA), the presumptive antimalarial treatment of an entire population to clear the subclinical parasite reservoir, is a strategy to accelerate malaria elimination. We report a cluster randomised trial to assess the effectiveness of dihydroartemisinin-piperaquine (DP) MDA in reducing falciparum malaria incidence and prevalence in 16 remote village populations in Myanmar, Vietnam, Cambodia, and the Lao People’s Democratic Republic, where artemisinin resistance is prevalent.

**Methods and findings:**

After establishing vector control and community-based case management and following intensive community engagement, we used restricted randomisation within village pairs to select 8 villages to receive early DP MDA and 8 villages as controls for 12 months, after which the control villages received deferred DP MDA. The MDA comprised 3 monthly rounds of 3 daily doses of DP and, except in Cambodia, a single low dose of primaquine. We conducted exhaustive cross-sectional surveys of the entire population of each village at quarterly intervals using ultrasensitive quantitative PCR to detect *Plasmodium* infections. The study was conducted between May 2013 and July 2017. The investigators randomised 16 villages that had a total of 8,445 residents at the start of the study. Of these 8,445 residents, 4,135 (49%) residents living in 8 villages, plus an additional 288 newcomers to the villages, were randomised to receive early MDA; 3,790 out of the 4,423 (86%) participated in at least 1 MDA round, and 2,520 out of the 4,423 (57%) participated in all 3 rounds. The primary outcome, *P*. *falciparum* prevalence by month 3 (M3), fell by 92% (from 5.1% [171/3,340] to 0.4% [12/2,828]) in early MDA villages and by 29% (from 7.2% [246/3,405] to 5.1% [155/3,057]) in control villages. Over the following 9 months, the *P*. *falciparum* prevalence increased to 3.3% (96/2,881) in early MDA villages and to 6.1% (128/2,101) in control villages (adjusted incidence rate ratio 0.41 [95% CI 0.20 to 0.84]; *p =* 0.015). Individual protection was proportional to the number of completed MDA rounds. Of 221 participants with subclinical *P*. *falciparum* infections who participated in MDA and could be followed up, 207 (94%) cleared their infections, including 9 of 10 with artemisinin- and piperaquine-resistant infections. The DP MDAs were well tolerated; 6 severe adverse events were detected during the follow-up period, but none was attributable to the intervention.

**Conclusions:**

Added to community-based basic malaria control measures, 3 monthly rounds of DP MDA reduced the incidence and prevalence of falciparum malaria over a 1-year period in areas affected by artemisinin resistance. *P*. *falciparum* infections returned during the follow-up period as the remaining infections spread and malaria was reintroduced from surrounding areas. Limitations of this study include a relatively small sample of villages, heterogeneity between villages, and mobility of villagers that may have limited the impact of the intervention. These results suggest that, if used as part of a comprehensive, well-organised, and well-resourced elimination programme, DP MDA can be a useful additional tool to accelerate malaria elimination.

**Trial registration:**

ClinicalTrials.gov NCT01872702

## Abstract 2

In a cluster-randomized trial, Lorenz von Seidlin & colleagues investigate whether mass drug administration can accelerate malaria elimination in the Greater Mekong Subregion.

## Author summary

**Why was this study done?:**

- The emergence and spread of multidrug resistance in the Greater Mekong Subregion (GMS) threaten regional and global malaria control.
- Mass drug administrations (MDAs) are controversial but could be useful in the control and elimination of malaria.
- We wanted to know whether well-resourced MDAs can accelerate malaria elimination in the GMS.

**What did the researchers do and find?:**

- We randomised 16 villages (clusters) to receive MDAs with antimalarial drugs (dihydroartemisinin-piperaquine [DP] plus low-dose primaquine) either in year 1 or year 2 of the study. The entire village population (except pregnant women and children under the age of 6 months) was invited to take 3 consecutive daily doses of antimalarial drugs 3 times at monthly intervals. Everyone was followed up for 1 year; all malaria cases were recorded, and quarterly malaria surveys were conducted using highly sensitive high-volume PCR detection.
- Most (87%) of the villagers completed at least 1 round of the antimalarial drugs, which were well tolerated.
- The intervention had a substantial impact on the prevalence of *P*. *falciparum* infections by month 3 after the start of the MDAs. Over the subsequent 9 months, *P*. *falciparum* infections returned but stayed below baseline levels.

**What do these findings mean?:**

- MDAs might be a useful tool to accelerate falciparum malaria elimination in low-endemicity settings.
- The effectiveness of MDAs depends on continued support for village health workers, adequate drug efficacy, high levels of community participation, and carefully planned roll out to minimise the risk of malaria reintroduction.

## Introduction

Considerable advances in malaria control and elimination have been achieved globally over the last decade. Since 2010 several former malaria endemic countries have been certified malaria-free. These include Sri Lanka, which had a high malaria burden (>100,000 cases/annually) at the beginning of the century while suffering from the consequences of a 25-year civil war [1]. Such success stories show that a determined malaria control programme with widespread use of long-lasting insecticide-treated bednets, insecticide spraying where appropriate, early diagnosis, and effective treatment can control and eliminate malaria. However, these conventional control tools are failing in some areas. Susceptibility of malaria vectors to most insecticides has decreased, often markedly, over the last decade [2], while the first-line treatments for *P*. *falciparum* malaria, artemisinin combination therapies (ACTs), are losing their efficacy in the Greater Mekong Subregion (GMS), home to more than 300 million people [3–6]. This is particularly worrying as resistance against earlier first-line antimalarial treatments (chloroquine, sulphadoxine-pyrimethamine) started in the GMS, spread to India and then to Africa, and killed millions of children [7]. More recently, parasites with resistance to both artemisinin and piperaquine emerged in western Cambodia and then spread to neighbouring countries [8,9]. Mefloquine resistance has re-emerged on the Thailand–Myanmar border. The decline in the effectiveness of the current first-line malaria drugs leaves few treatment options for falciparum malaria in the GMS. The spread of ACT-resistant *P*. *falciparum* strains into sub-Saharan Africa could become a public health emergency. Stopping the spread of antimalarial resistance requires the interruption of *P*. *falciparum* transmission.

Mass drug administrations (MDAs) clear symptomatic infections and, critically, also asymptomatic infections, which otherwise escape detection. MDAs may be essential to stop transmission and speed up the elimination of malaria. MDAs have been a part of the malaria control armamentarium for more than 100 years [10]. Three major reviews of MDAs have been conducted [10–12], which found that MDAs could interrupt malaria transmission temporarily in several areas and were critical for the permanent elimination of malaria from islands in the Pacific Ocean [13]. The success of MDAs depends on the efficacy of the drug regimen, the coverage of the target population, and the local malaria epidemiology—in particular the sources of transmission and potential for re-importation of malaria. MDAs have generally provided only transient benefit in areas of higher transmission because of rapid reintroduction of malaria from surrounding areas, and their role has remained controversial [12,14–16]. Targeted malaria elimination (TME) combines MDA with ensuring access to long-lasting insecticide-treated bednets and provision of early diagnosis and appropriate treatment. How long TME can interrupt or reduce the transmission of *P*. *falciparum* infections in communities in the GMS with low but persistent malaria transmission is not known.

**Fig 1:**
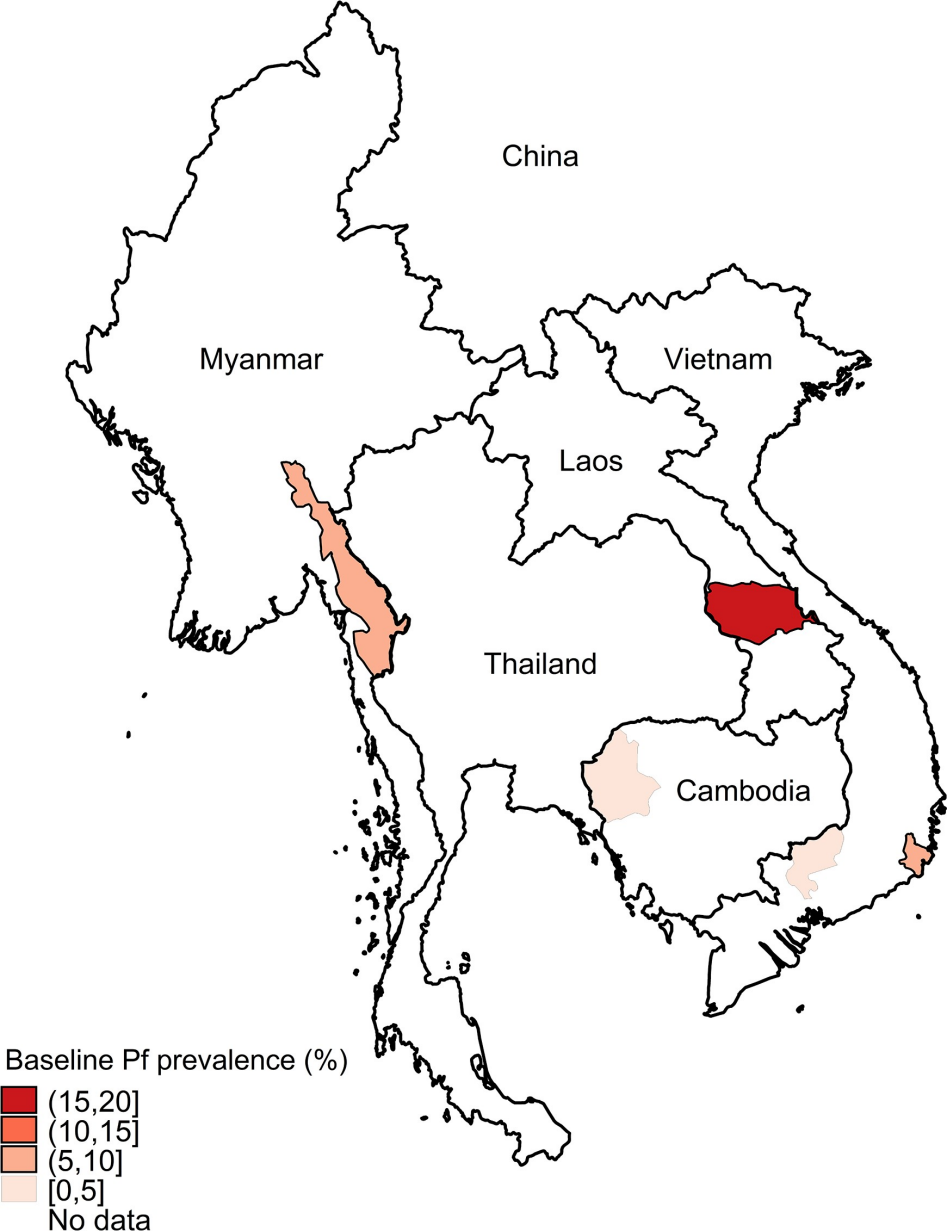
Countries and study sites in the Greater Mekong Subregion. Baseline *P*. *falciparum* (Pf) prevalence is shown for the 5 study sites (1 in each country except Vietnam, which had 2).

We performed a cluster randomised trial in Myanmar, Vietnam, Cambodia, and People’s Democratic Republic (Lao PDR) (Fig 1), where malaria transmission is generally low (entomological inoculation rates < 1 infective bite/person/year). Malaria transmission occurs all year but increases during the rainy season, which lasts from June to October in Myanmar, May to November in Vietnam, and May to October in Lao PDR and Cambodia [17–20]. This article describes the impact of MDA on *P*. *falciparum* infections; the impact on *P*. *vivax* infections will be the subject of a subsequent report.

## Methods

The MDAs started on 27 May 2013 in the sites in Myanmar, followed by Vietnam on 11 November 2013, Cambodia on 21 July 2015, and Lao PDR on 21 April 2016, and the study ended with the completion of follow-up of the Lao PDR villages on 12 June 2017 (S2 Table). There was a 12-day delay between the start of the study on 27 May 2013 and the public registration of the trial on 7 June 2013 due to an administrative delay.

### Study sites

#### Myanmar

In response to large numbers of malaria cases in eastern Myanmar, the Shoklo Malaria Research Unit installed and supported health posts with facilities for malaria diagnosis and treatment in several villages (populations 300 to 800 people) in 2013. This programme is ongoing and by 2018 had expanded to include more than 1,200 villages in rural eastern Kayin (Karen) State of Myanmar [21]. The health posts were focal points for cross-sectional surveys in 12 villages conducted to plan control interventions [22]. Based on the findings from these surveys, 4 villages located within 10 km of the Thailand border were selected, considered representative of the region in terms of environment, ecology, malaria epidemiology, population, and human behaviour. Artemisinin resistance in *P*. *falciparum* is well established in this area. Findings from this pilot study in eastern Myanmar have been published [23].

#### Vietnam

There has been a substantial reduction in the incidence of malaria in Vietnam over the last 20 years, but malaria still remains a public health challenge in some rural areas [24]. Investigators from the Oxford University Clinical Research Unit selected 2 villages in Binh Phuoc Province and 2 villages in Ninh Thuan Province in the south-central region for the study, based on previously acquired data from surveys in 12 villages [22]. Since 2010, studies in Binh Phuoc Province have shown an increasing proportion of artemisinin-resistant infections. At the time of the site selection in 2014, there was no evidence of piperaquine resistance in Binh Phuoc, and cure rates with the first-line treatment dihydroartemisinin-piperaquine (DP) remained satisfactory [3,25]. Multidrug resistance was first detected in Binh Phuoc in 2016 [9], and treatment failures with DP have risen substantially since then [26].

#### Cambodia

Artemisinin-resistant *P*. *falciparum* was first confirmed in Pailin in western Cambodia more than 10 years ago [27,28]. Cambodia used DP as first-line treatment until 2016, by which time failure rates had increased markedly, particularly in the west of the country. This prompted a change in first-line antimalarial treatment policy to artesunate-mefloquine, although provision of the drug lagged behind [9]. Many containment efforts in Cambodia have focused on Pailin, and these have resulted in a marked decline in malaria incidence in the province [29–33]. Surveys in 20 western Cambodian villages by the Mahidol Oxford Research Unit and the Cambodian National Malaria Control Programme showed low prevalences of subclinical *P*. *falciparum* infections, which were nearly all in villages near forested areas [34]. Four forest-fringe villages along the Thai border in Battambang Province, adjacent to Pailin Province were included in the present study [35].

#### Lao PDR

The study was conducted in southern Savannakhet Province, Lao PDR, which historically has been one of the regions with the highest malaria prevalence in the country. Artemether-lumefantrine is the first-line treatment for falciparum malaria [36]. Investigators from the Lao–Oxford–Mahosot Hospital–Wellcome Trust Research Unit conducted cross-sectional surveys in 18 villages in Thapangthong and Nong districts of Savannakhet Province based on access and previously reported high malaria incidences in provincial epidemiological records [37]. Based on the findings from the surveys, the investigators selected 4 villages in Nong district for the study.

### Censuses

In each site study teams performed a baseline census. The study teams geo-localised all houses by GPS and assigned a unique identification number linked to the study number of each household member. The census recorded the de jure population in each village, which tallies people according to their regular or legal residence. During subsequent surveys, the study teams recorded the de facto population, i.e., the number of people sleeping in the village during the night before the survey.

### Community engagement, incentives, and ancillary care

At each site, formative research conducted in preparation for the study suggested that, in addition to the information content, the background of the person providing information was a critically important determinant of the effectiveness of the communications [38–43]. Establishing trust between community members and the study team was a key prerequisite for a functioning relationship with the study populations. Team members who resided in the village for extended periods were encouraged to participate in local life and community events, such as weddings, funerals, and festivals. Participation in daily community life allowed the team to develop an understanding of local hierarchies, the seasonality of village work, and the needs and preferences of community members [44,45].

To increase the uptake and thus impact of MDAs, the study teams conducted intensive community engagement at each study site, starting from the time of site selection. This included educational elements to ensure that every community member was informed about the purpose of the MDA and the need for the entire village to participate to maximise individual benefits, and education to allay fears about risks of the antimalarial drugs. Village leaders, village malaria workers, and volunteers formed committees that assisted the study teams in organising the survey and designing and implementing community engagement. The teams also organised community engagement meetings at different levels, from individual or household informal discussions to whole village assemblies. Children and young people were engaged through activities such as music festivals and theatre.

Study teams provided benefits and incentives for participation at all sites (S1 Table). They adapted incentives according to the wishes of the villagers, as discussed in community meetings, and the preferences of regulatory authorities. In some sites, the study teams provided individual cash or non-cash incentives combined with gifts and/or lottery tickets, whereas in other sites the study teams preferred community incentives, e.g., improved water supply for the entire village. The study teams provided basic primary healthcare in the study villages. This ancillary care was essential to establish trust between the study team and community members. Health education on topics unrelated to MDA, such as family planning, nutrition, and vaccinations, was provided to community members at their request. The research teams worked to ensure that all participating villages had uninterrupted access to early diagnosis, efficacious antimalarial treatment, and insecticide-treated bednets.

### Mass drug administrations

The primary target of the MDAs was falciparum malaria. Radical cure of latent *P*. *vivax*, which would consist of a 7- to 14-day course of the 8-aminoquinoline primaquine, was not provided. The study teams conducted DP MDAs at month 0 (M0), M1, and M2 in early (intervention) MDA villages, whereas deferred (control) MDAs were conducted at M12, M13, and M14 in all sites except for the study sites in Myanmar, where, for operational reasons—specifically the limited access to study sites during the anticipated heavy rains—the deferred MDAs were conducted at M9, M10, and M11 (S1 Fig). In each country we matched the village pairs by geographical proximity, population size, and parasite prevalence. In each pair we selected 1 village randomly to receive early MDA and the other village to receive deferred MDA [46]. The randomisation was based on computer-generated random numbers provided by the trial statistician. For each round of treatment, the study team set up a mobile clinic or specific malaria post. Participants received MDA at home if they were unable to come to the clinic. The drug regimen comprised 3 rounds of 3 daily doses of DP combined with a single low dose of primaquine (0.25 mg/kg) given at intervals of 1 month. For regulatory reasons, no primaquine was administered in Cambodia. One DP tablet contained 40 mg of dihydroartemisinin and 320 mg of piperaquine. A weight-based regimen aiming at a total dose of 7 mg/kg dihydroartemisinin and 55 mg/kg piperaquine phosphate was used (Eurartesim, Sigma Tau, Italy, or Guilin Pharmaceutical, Guilin, China). The study teams encouraged all residents in the study villages to take part in the drug administration except for children under the age of 6 months and pregnant women (first trimester pregnancies were excluded in Myanmar and Vietnam and all pregnant women were excluded in Cambodia and Lao PDR). All doses were administered under direct observation of the study teams. All drugs were provided with snacks to increase tolerability. For children unable to swallow the tablets, the staff crushed the tablets and administered them mixed with water. Newcomers to the village or returning residents arriving during the study period were contacted by village malaria workers and offered a single round of DP. No placebo was used in control villages.

The rationale for the treatment regimen has been described previously [47]. Briefly, a full 3-day treatment dose of DP was given in order to clear any *P*. *falciparum* infection. The rationale for 3 repeated monthly rounds (3× 3 doses) was to provide the 3 months of post-treatment prophylactic effect needed to interrupt malaria transmission. This period was considered necessary because infectious mosquitos can survive for 30 days, and some community members were absent during the drug administration (they were treated during subsequent rounds) [48–51]. A single low dose of primaquine is sufficient to sterilise and rapidly clear mature *P*. *falciparum* gametocytes, which are not susceptible to schizonticidal drugs [50].

### Tolerability and adverse event monitoring

The study teams observed participants for 1 hour after drug administration and in case of vomiting offered a full or half repeat dose depending on the interval since the primary administration. Study staff enquired about adverse events (AEs) using a structured questionnaire on days 2, 3, and 7, and again 1 month after drug administration. During the 3-month intervention, a mobile clinic staffed with a medical assistant was available in the village to provide free consultations to villagers and to assess participants presenting with AEs. All serious AEs (SAEs) including hospitalisations and deaths occurring within 3 months of the first drug administration were documented and investigated by the study team, and the association with treatment was assessed.

### Surveillance and follow-up

The surveillance period was 24 months in the study sites in Myanmar and Vietnam but stopped after 12 months in Cambodia and Lao PDR because of the delayed starts in Cambodia and Lao PDR (see also S1 Fig). Just before the MDA and then every 3 months after the MDA, the study teams invited all people residing in the study villages aged 6 months or older, including temporary residents and migrant workers arriving after the MDA, to participate in the cross-sectional prevalence surveys. The survey at M21 in Myanmar was omitted because access to the study sites became impossible for the study teams during heavy rains. At the quarterly surveys, the study teams recorded the presence or absence of each participant in the village during the preceding 3 months. Additional information was collected during home visits at 2-weekly intervals: a study staff member enquired about the presence of recorded household members. Study staff recorded all newcomers who intended to stay in the village for longer than 2 weeks. Demographic information was collected, and participants’ temperature (skin surface forehead or tympanic membrane), weight, and height were measured by the study team. The threshold for determining fever was 38°C for body surface temperature and 37.5°C for tympanic temperature. A rapid diagnostic test (RDT) was performed, and if the test was positive for malaria, patients were treated according to national guidelines. The study team collected venous blood (3 ml from all individuals aged ≥5 years, and 500 μl from children aged ≥6 months to 5 years) at each survey, and from any individuals who developed fever during the study period.

### Laboratory investigations

The study team stored blood samples in a cool box in the field and transported the samples within 12 hours to the local laboratory. The teams tested blood samples from all survey participants using standard microscopy and malaria RDTs (Myanmar, Lao PDR, and Vietnam: SD Bioline Malaria Ag P.f/Pan POCT, Standard Diagnostics, Yongin-si, Republic of Korea; Cambodia: Healgen Malaria *P*. *falciparum*/Pan 1-step RDT, Zhejiang Orient Biotech, China). Microscopists who had at least 5 years’ experience and/or were confirmed to be Level 2 or higher, as assessed by a standard WHO 55 slide set, performed the standard microscopy, counting the number of parasites per 500 white blood cells on Giemsa-stained peripheral blood thick films. After separation of plasma, buffy coat, and packed red blood cells, samples were frozen and stored at −80°C. The study teams transported frozen samples from Myanmar, Cambodia, and Lao PDR monthly on dry ice to the molecular laboratory in Bangkok, Thailand, and the samples from the Vietnam sites to Ho Chi Minh City, Vietnam, for DNA extraction and high-volume ultrasensitive quantitative PCR (uPCR).

### *Plasmodium* detection

We have previously reported a detailed description and evaluation of the uPCR methods [52]. In summary, we purified the DNA template for PCR detection and quantification of *Plasmodium* from the thawed packed red blood cell samples. The purified DNA was dried completely in a centrifugal vacuum concentrator and then suspended in a small volume of PCR grade water, resulting in a concentration factor defined by the original blood volume (100–2,000 μl) divided by the resuspended double distilled water volume (10–50 μl). We used 2 μl of resuspended DNA as template in the quantitative PCR reaction. We assessed the presence of malaria parasites and estimated the parasite load in each sample using an absolute quantitative real-time PCR method. The 18S rRNA–targeting primers and hydrolysis probes used in the assay have been validated and are highly specific for *Plasmodium* species [53]. The lower limit of accurate quantitation using this method is 22 parasites per millilitre of whole blood. We used a QuantiTect Multiplex PCR NoROX Kit (Qiagen, Hilden, Germany) in the Bangkok laboratory and an absolute quantitative real-time PCR (quantitative PCR) method (Roche, Basle, Switzerland) in the laboratory in Ho Chi Minh City. We determined the *Plasmodium* species in uPCR positive samples using nested PCR specific to *P*. *falciparum* (microsatellite marker Pk2), *P*. *vivax* (microsatellite marker 3.502), and *P*. *malariae* (18s rRNA) as described previously [53–55]. We reported positive samples for which there was insufficient DNA for species identification—or where no amplification was obtained in this step—as being of indeterminate species.

### Detection of molecular markers of antimalarial resistance

We assessed polymorphisms in the *PfKelch13* gene by nested PCR amplification covering the full length of the gene (total 2,181 bp) and sequenced the gene by ABI Sequencer (Macrogen, Seoul, Republic of Korea) as described previously. We monitored cross-contamination by adding negative control samples in every run. Sequencing results were aligned against *PfKelch13* of reference strain 3D7 (putative 9PF13_0238 NCBI Reference Sequence [3D7]: XM_001350122.1), using Bioedit software (Abbott, Santa Clara, CA, US). Two study technicians assessed polymorphic patterns blinded to the origin of the sample.

We quantified *Pfplasmepsin2/3* gene copy number using relative quantitative real-time PCR based on Taqman probe on a Corbett Rotor-Gene Q (Corbett Research, Mortlake, NSW, Australia). Primers and probes have been described previously [56]. We performed amplification in triplicate on a total volume of 25μl as multiplex PCR using a QuantiTect Multiplex PCR NoROX Kit (Qiagen, Hilden, Germany). Every amplification run contained 9 replicates of calibrators and triplicates without template as negative controls. *Plasmodium-*specific beta-tubulin served as an internal standard for the amount of sample DNA added to the reactions.

### Analysis

We categorised each resident’s individual MDA exposure as (a) did not participate at all, (b) did not complete a single round (3 doses), (c) completed only 1 round, (d) completed only 2 rounds, or (e) completed all 3 rounds. For the estimation of MDA coverage, we defined the numerator as the number of participants during three MDA rounds and the denominator as the de facto population during the time of MDA rounds. We defined *Plasmodium* prevalence by the uPCR result, but in the absence of a uPCR result we considered a positive microscopy or RDT result as sufficient to classify an individual as infected.

A *P*. *falciparum* infection was defined by either a *P*. *falciparum* positive result or a mixed result of *P*. *falciparum* and *P*. *vivax*. The incidence was defined using the number of malaria infections as numerator and exposure time as denominator. The individual exposure time was defined as the number of days spent within the catchment area, i.e., the village and the surrounding farms. The exposure time was estimated in 3-month intervals. For example, if a resident was present during 2 sequential surveys, the exposure time was 90 days. If a resident was missing during a survey, we assumed he/she stayed in the village for 45 days after the last participation in a survey. Similarly, we assumed a new arrival had arrived 45 days before the first participation in a survey. We assumed both losses to follow-up and intermittent missing data were missing at random. Seasons were defined as wet or dry by country as described above.

The unit of randomisation and hence the unit of statistical inference was the village cluster. The primary approach to analysis was based on intention to treat (ITT). In order to assess the sensitivity of our assumptions in the ITT approach, we also performed a dose-related per protocol analysis. We compared changes in prevalence then in incidence of *P*. *falciparum* (including mixed *P*. *vivax* and *P*. *falciparum*) infections over 12 months between villages that received early MDA and those that received deferred MDA.

Before the data collection was completed we drafted a statistical analytic plan, which is included as S1 Text. We examined the impact of DP MDA on malaria using multilevel mixed-effects Poisson models to obtain incidence rate ratios (IRRs) of *Plasmodium* infections. For the multilevel models, level 1 was repeated measurements of villagers over the follow-up time, level 2 was participants from the same village, level 3 was the 16 randomised villages, level 4 was the 4 different countries.

First, we performed univariable analyses to obtain the unadjusted estimates of IRRs of the association between malaria infections and MDA status, followed by adjustment for variables prespecified in the statistical analytic plan that are predictive for outcome and potentially imbalanced, i.e., sex, age, fever, bednet use, season, and prevalence of *P*. *falciparum* infections in the village. In an alternative model we used MDA exposure, i.e., 0, 1, 2, or 3 completed rounds as the main independent categorical exposure variable. In the initial analysis, we included only the exposure time from the completion of the MDA at M3 to M12. In a secondary analysis, we included the period from M0 to M12, i.e., including the MDA implementation period. For the analyses reported here, focusing on *P*. *falciparum*, we excluded parasitaemias in which the species could not be identified (indeterminate species, *Plasmodium* spp.). As the lower limit of quantification is 22 parasites/ml, genome densities less than 22 parasites/ml (*n =* 95 participants at baseline) were not included in the analysis, but the infection status (infected or uninfected) was left unchanged. The control villages in Myanmar, which received MDA at M9 during the crossover period, were excluded from the analysis at M12. We provide the intra-cluster correlation coefficient (ICC) for the incidence of *P*. *falciparum* infections with village (cluster) as a unit of randomisation, accounting for the random effect of country, using the exact linearisation calculation approach [57].

The sample size, 4 village clusters per country, was chosen mainly for operational and practical reasons. A formal sample size calculation suggested that 16 villages would provide 80% power to detect a 95% fall in prevalence from a 10% initial prevalence, controlling for random changes in prevalence in the control groups, with a minimum of at least 152 individuals in each village recruited and followed up satisfactorily.

The study teams collected survey data on case record forms and entered the data on smartphones before exporting them into OpenClinica (OpenClinica, Waltham, MA, US). Graphical summaries have been presented to show prevalence and incidence patterns over time. Treatment and AE data were recorded on registers and then entered in Excel (Microsoft, Redmond, Washington, US). Analyses were performed in STATA 15.0 (StataCorp, College Station, Texas, US).

### Ethics approvals

The studies were approved by the Cambodian National Ethics Committee for Health Research (0029 NECHR, dated 04 Mar 2013), the Institute of Malariology, Parasitology, and Entomology in Ho Chi Minh City (185/HDDD, dated 15 May 2013), the Institute of Malariology, Parasitology, and Entomology in Quy Nhon (dated 14 Oct 2013), the Lao National Ethics Committee for Health Research (Ref No 013-2015/NECHR), the Government of the Lao PDR, and the Oxford Tropical Research Ethics Committee (1015–13, dated 29 Apr 2013). Each participant, or parent/guardian in the case of minors, provided individual, signed, informed consent; illiterate participants provided a fingerprint countersigned by a literate witness (ClinicalTrials.gov Identifier: NCT01872702).

## Results

**Fig 2:**
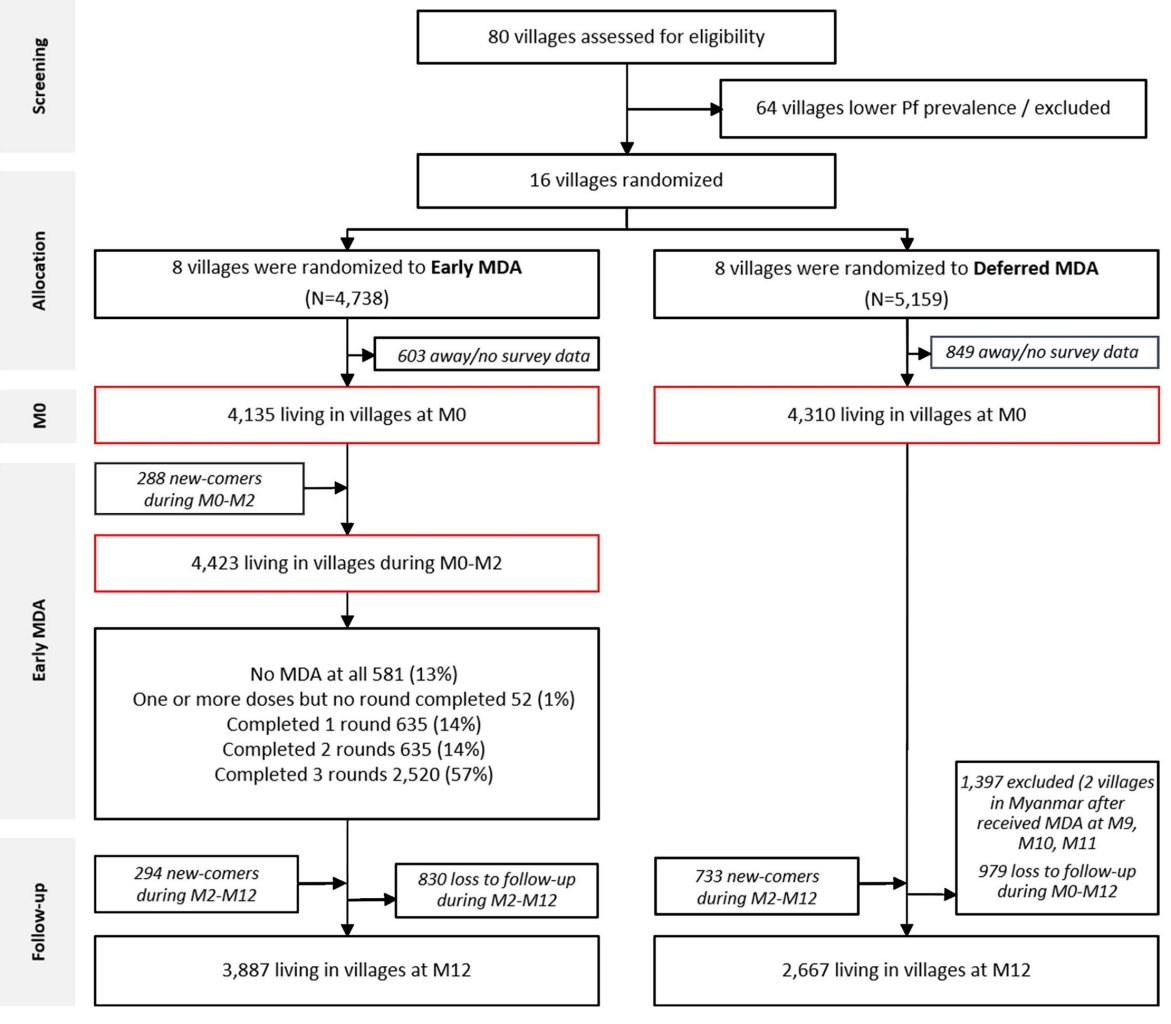
CONSORT flow chart—the first 12 months. M[number], month [number]; MDA, mass drug administration; Pf, *P*. *falciparum*.

The de jure population in the 16 villages was 9,897 (4,738 in early MDA and 5,159 in deferred MDA villages). The de facto population at M0 was 8,445 (4,135 in early MDA and 4,310 in deferred MDA villages), with a median of 495 residents per village (Fig 2). The median age of participants was 20 years (interquartile range 9 to 36). The large majority reported using insecticide-treated bednets regularly (4,579/5,620; 82%; Table 1). At baseline (M0), uPCR detected an overall mean *P*. *falciparum* prevalence of 6.2% (95% CI 5.6% to 6.8%). The baseline *P*. *falciparum* prevalence was lower in early MDA villages (5.1%, 95% CI 4.4% to 5.9%) compared to villages with deferred MDA (7.2%, 95% CI 6.4% to 8.1%), as was the *P*. *falciparum* density: geometric mean 3,363 parasites/ml (95% CI 2,472 to 4,575) in early MDA villages compared with 10,607 parasites/ml (8,146 to 13,812) in villages assigned to deferred MDA.

**Table 1:** Baseline characteristics of participants immediately before mass drug administration.

Characteristic	Control (deferred MDA) villages	Intervention (early MDA) villages	Overall
People living in villages at month 0, *n*	4,310	4,135	8,445
Sex, *n* (%)			
Male	2,175 (50.5)	2,099 (50.8)	4,274 (50.6)
Female	2,135 (49.5)	2,036 (49.2)	4,171 (49.4)
Age (years), median (IQR)	19 (8, 36)	21 (9, 37)	20 (9, 36)
Weight (kg), median (IQR)	41 (21, 51)	44 (24, 52)	43 (22, 51)
Height (cm), median (IQR)	148 (123, 156)	148 (127, 156)	148 (124, 156)
Temperature (°C), median (IQR)	36.8 (36.6, 37.1)	36.9 (36.6, 37.1)	36.9 (36.6, 37.1)
Fever, *n* (%)	*n =* 2,889	*n =* 3,337	*n =* 6,226
Yes	393 (13.6)	462 (13.8)	855 (13.7)
No	2,496 (86.4)	2,875 (86.2)	5,371 (86.3)
Bednet use, *n* (%)	*n =* 2,816	*n =* 2,804	*n =* 5,620
Regular	2,377 (84.4)	2,202 (78.5)	4,579 (81.5)
Irregular	364 (12.9)	495 (17.7)	859 (15.3)
Never use	75 (2.7)	107 (3.8)	182 (3.2)
Baseline infection	*n =* 3,405	*n =* 3,340	*n =* 6,745
*Pf* infection, *n* (%, 95% CI)	246 (7.2, 6.4–8.1)	171 (5.1, 4.4–5.9)	417 (6.2, 5.6–6.8)
*Pv* infection, *n* (%, 95% CI)	405 (11.9, 10.8–13.0)	288 (8.6, 7.7–9.6)	693 (10.3, 9.5–11.0)
*Pf* uPCR genomes/ml, geometric mean (95% CI)	10,607 (8,146–13,812)	3,363 (2,472–4,575)	6,443 (5,260–7,892)

CI, confidence interval; IQR, interquartile range; MDA, mass drug administration; *Pf*, *P*. *falciparum*; *Pv*, *P*. *vivax*; uPCR, ultrasensitive quantitative PCR.

### MDA coverage

Of the 4,423 people residing during M0, M1, and M2 in the 8 villages randomised to early MDA, 3,790 (86%) completed at least 1 round (3 doses) of DP MDA: 635 (14%) completed 1 round of antimalarials, 635 (14%) completed 2 rounds, and 2,520 (57%) completed all 3 rounds (Fig 2). Thus, 633 residents (14%) did not complete a single round or took no antimalarials at all. In all, 2,707 residents lived in the 4 control villages in Myanmar and Vietnam where 24-month follow-up was conducted. Of these, 2,185 (81%) completed at least 1 round of MDA: 530 (20%) completed 1 round of antimalarials, 618 (23%) 2 rounds, and 1,037 (38%) all 3 rounds. Thus, 522 residents (19%) did not complete a single round or took no antimalarials at all (S2 Fig). In total, of the 8,749 study participants present during any time of the study period, 5,848 (67%) participated in at least 3 of the 5 possible surveys, and 2,815 (32%) in all 5 surveys (Table S2). The start and end date of each MDA is listed in S2 Table. The participation in follow-up surveys is shown in S3 Table.

### *P*. *falciparum* prevalence and incidence

**Fig 3:**
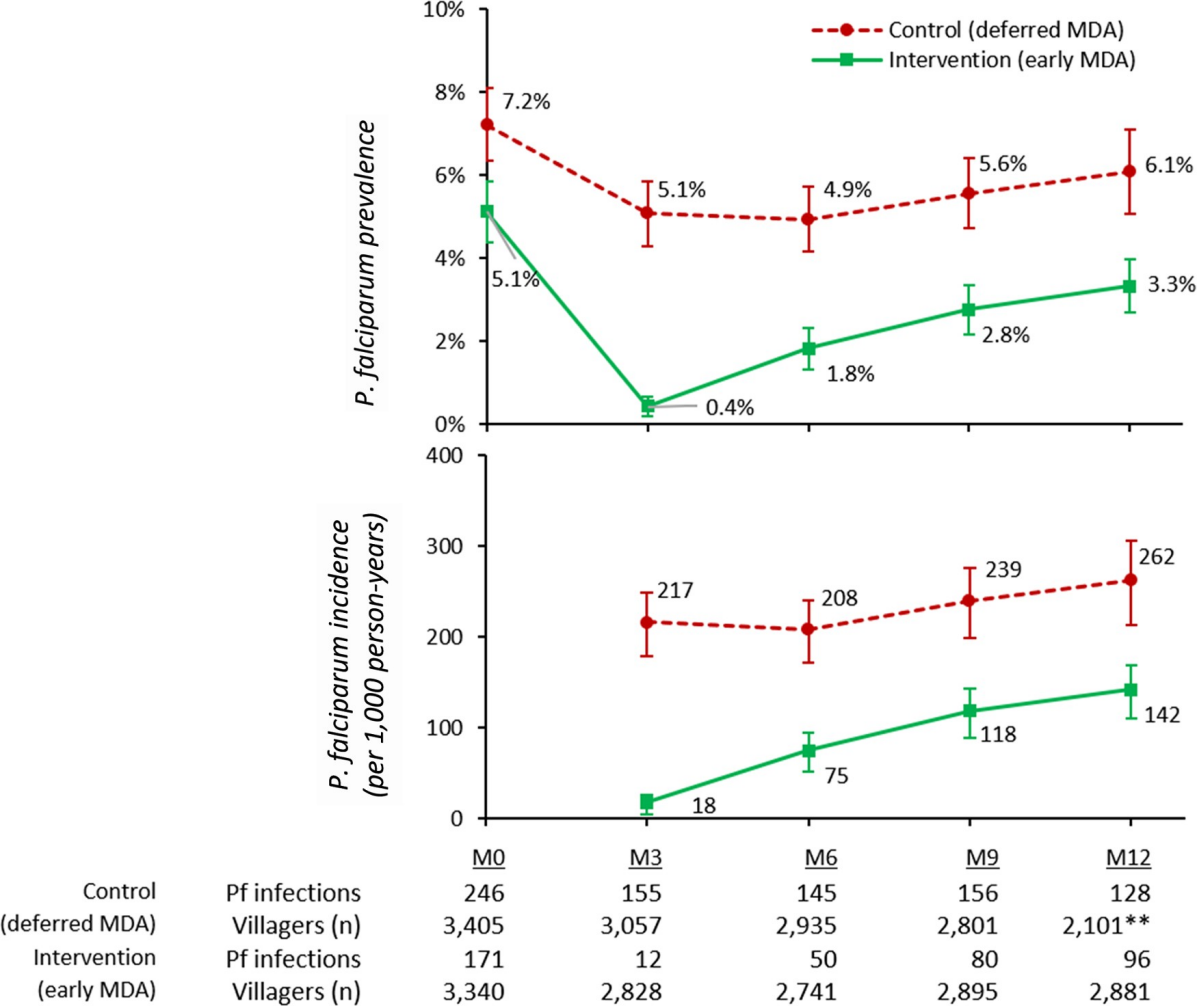
Prevalence and incidence of *P*. *falciparum* (with 95% confidence intervals) detected using ultrasensitive quantitative PCR in 8 intervention (early MDA) and 8 control (deferred MDA) villages over a 12-month follow-up period. M[number], month [number]; MDA, mass drug administration; Pf, *P*. *falciparum*.

Three months (M3) after the first round of drug administrations, the *P*. *falciparum* prevalence in the early MDA villages had fallen by 92%, from 5.1% (171/3,340) to 0.4% (12/2,828), while in control villages the *P*. *falciparum* prevalence had decreased by 29%, from 7.2% (246/3,405) to 5.1% (155/3,057; difference in differences −2.5%, 95% CI −3.9 to −1.1%, *p* < 0.001; Fig 3). The *P*. *falciparum* prevalence rose steadily over the following 9 months in the villages that received early MDA, to 3.3% (96/2,881) at M12, a 7-fold increase. The *P*. *falciparum* prevalence in the control villages, which received deferred MDA, rose from 5.1% (155/3,057) to 6.1% (128/2,101) during the same period, a 20% increase. The *P*. *falciparum* incidence after early MDA was 18 infections per 1,000 person-years and in control villages (deferred MDA) was 217 per 1,000 person-years (*p* < 0.001). Over the following 9 months, the *P*. *falciparum* incidence rose in early MDA villages to 142 per 1,000 person-years and in villages with deferred MDA to 262 per 1,000 person-years. There were 10 cases of acute falciparum malaria in intervention villages and 12 in control villages during the surveillance period.

Following the crossover, MDA surveillance continued for a further 12 months in 8 villages in Myanmar and Vietnam (S3 Fig). Following the deferred MDAs in Myanmar and Vietnam, the *P*. *falciparum* prevalence fell by 90% over 6 months, from 6.1% (128/2,101) to 0.6% (10/1,575), while in villages that had received early MDA during the previous year (but none in the current year), *P*. *falciparum* prevalence nearly doubled over 3 months, from 3.3% (96/2,881) at M12 to 6.1% (90/1,483) at M15. It fell to 3.3% (44/1,350) at M18 and then stabilised between 3.3% (44/1,350) and 3.5% (54/1,549) between M18 and M24. The incidence of *P*. *falciparum* infections also fell by 90% in the 6 months after the deferred MDA, from 262 to 27 per 1,000 person-years, while in villages that had received early MDA during the previous year, the *P*. *falciparum* incidence rose from 142 to 261 per 1,000 person-years, then dropped to 77 per 1,000 person-years by M24. The overall impact of MDA in reducing the incidence of *P*. *falciparum* infections was highly significant. The adjusted IRR was 0.41 (95% CI 0.20 to 0.84) over the 9 months following implementation (Table 2).

**Table 2:** Multilevel mixed-effects Poisson regression on *P*. *falciparum* infections detected by ultrasensitive quantitative PCR during follow-up (month 3 to month 12).

Characteristic	Univariable model		Multivariable model A: MDA intervention (ITT)		Multivariable model B: MDA coverage (dose-related PP)	
	IRR (95% CI)	*p*-Value	IRR (95% CI)*	*p*-Value	IRR (95% CI)*	*p*-Value
Model A: Intervention						
Early MDA village	0.32 (0.12, 0.89)	0.029	0.41 (0.20, 0.84)	0.015		
Control village	Reference		Reference			
Model B: Coverage						
MDA completed 3 rounds	0.21 (0.08, 0.57)	0.002			0.30 (0.15, 0.58)	<0.001
MDA completed 2 rounds	0.46 (0.16, 1.31)	0.147			0.60 (0.29, 1.23)	0.160
MDA completed 1 round	0.73 (0.25, 2.15)	0.568			0.90 (0.42, 1.90)	0.777
MDA not completed/no MDA	0.81 (0.28, 2.30)	0.689			0.96 (0.47, 1.98)	0.918
Control village	Reference				Reference	
Sex						
Male	2.00 (1.64, 2.43)	<0.001	2.02 (1.66, 2.46)	<0.001	1.64 (1.42, 1.89)	<0.001
Female	Reference		Reference		Reference	
Age (years)	1.00 (0.99, 1.01)	0.072	1.01 (1.00, 1.01)	0.042	1.00 (0.99, 1.01)	0.300
Fever	1.40 (1.09, 1.80)	0.009	1.42 (1.10, 1.83)	0.007	1.42 (1.13, 1.79)	0.003
Bednet use						
Regular	Reference					
Irregular	1.36 (1.08, 1.71)	0.009				
No use	2.02 (1.37, 2.99)	<0.001				
Season						
Wet	1.06 (0.92, 1.22)	0.426	1.10 (0.96, 1.27)	0.187	1.07 (0.94, 1.23)	0.312
Dry	Reference		Reference		Reference	
Prevalence of *Pf* infection at baseline in village	1.12 (1.07, 1.17)	<0.001	1.10 (1.06, 1.15)	<0.001	1.09 (1.04, 1.14)	0.001

*Adjusted for all baseline variables except bednet use because missing data (35%; 7,801/22,239) substantially reduced the sample for complete case analysis.

IRR, incidence rate ratio; ITT, intention to treat; MDA, mass drug administration; *Pf*, *P*. *falciparum*; PP, per protocol.

### Heterogeneity in impact

**Fig 4:**
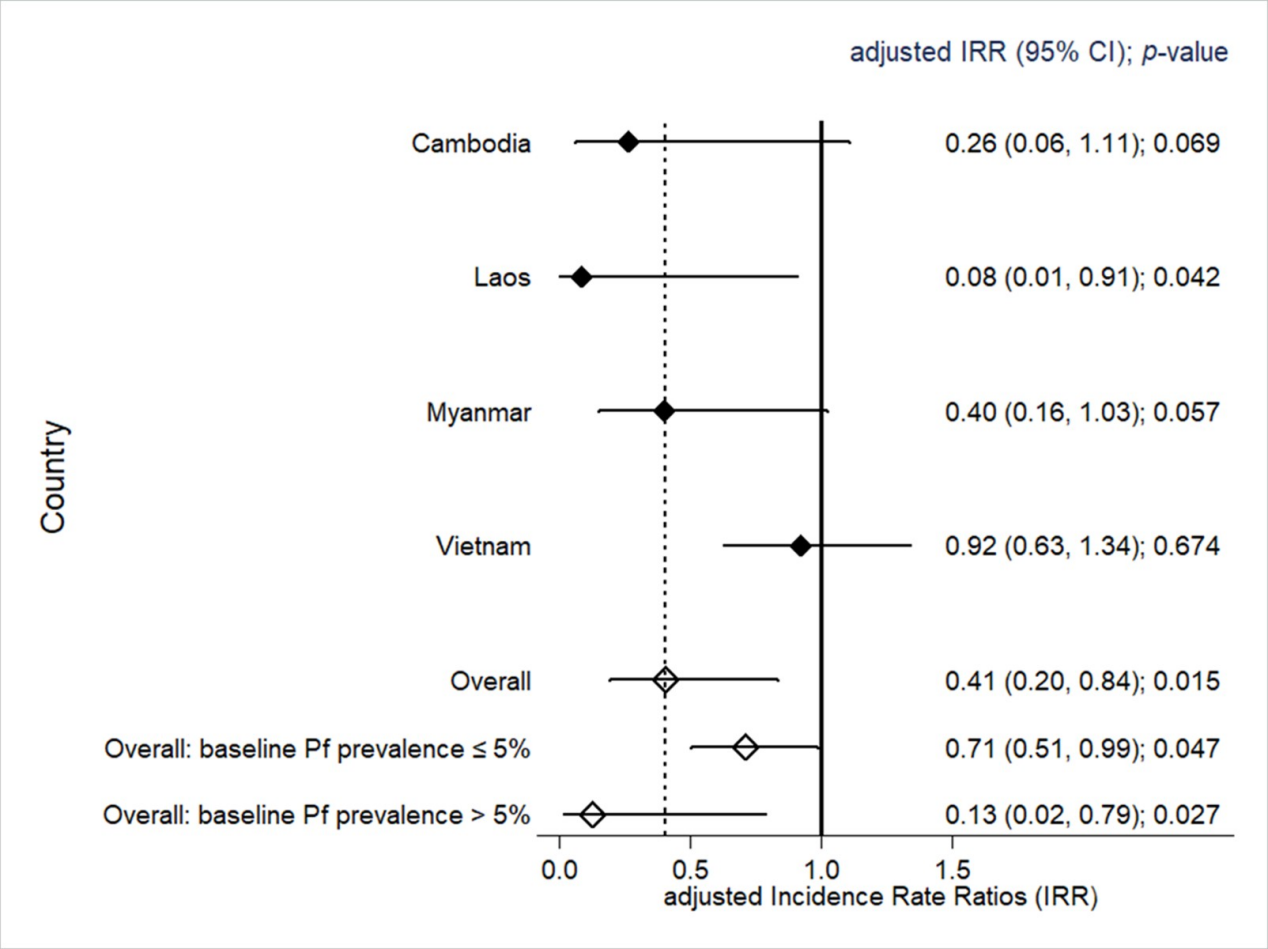
Comparison of incidence rate ratios of *P*. *falciparum* infections detected by ultrasensitive quantitative PCR between early MDA and deferred MDA villages, by country. MDA, mass drug administration; Pf, *P*. *falciparum*.

The impact of MDA on falciparum malaria varied by country. The greatest impact was in Lao PDR, followed by Cambodia and Myanmar, and there was little effect in Vietnam. This resulted in a country effect variance of 6.82 (*p =* 0.009; Fig 4). The impact was lower in villages with a baseline *P*. *falciparum* prevalence ≤ 5% (adjusted IRR 0.71, 95% CI 0.51 to 0.99) compared to villages with a baseline prevalence > 5% (adjusted IRR 0.13, 95% CI 0.02 to 0.79). The *P*. *falciparum* prevalence from 3 to 12 months after the MDA as assessed by uPCR was 0 or close to 0 (<1%) in 4 of 8 villages receiving early MDA and also in 4 of 8 control villages receiving deferred MDA (S4 Fig). The ICC for the incidence of *P*. *falciparum* infections for villages (as clusters), accounting for the random effect of country, was in the range from 0.06 to 0.32, estimated at baseline and every 3 months up to M12. The weighted-average ICC was 0.27 over 1 year of follow-up.

### Factors determining the impact of MDA on *P*. *falciparum* infections

In regression models that used village allocation of MDA as the main covariate, male sex, age, and presence of fever were each independently and significantly associated with *P*. *falciparum* infections (Table 2, model A). In models that replaced village allocation of MDA with MDA coverage (Table 2, model B), there was a highly significant dose–response relationship between protection and number of completed rounds (IRR 0.63, 95% CI 0.56 to 0.72, *p* < 0.001). Protection against *P*. *falciparum* infection was lowest in people who had not participated in the MDA and was reduced in people who took 1 or 2 doses but did not complete a single 3-dose round. Protection against *P*. *falciparum* infection was highest in participants who completed all 3 rounds of the 3-dose regimen. Models in which the observation period included M0 to M12 showed similar exposure–response relationships (S4 Table). In a model including respondents of all ages, the protection for *P*. *falciparum* infection increased significantly with regular bednet use. In a model that included only children under 12 years of age, no protection attributable to bednets could be detected (S5 Table). As data for bednet use was missing for 35% (7,801/22,239) of the total observations of participants, this variable was not included in the multivariable analyses.

### Parasite clearance and antimalarial resistance

**Fig 5:**
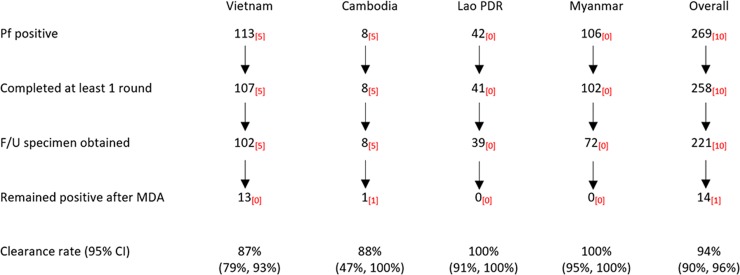
*P*. *falciparum* clearance after MDA: Dihydroartemisinin-piperaquine efficacy against asymptomatic infections estimated from individual-participant-level data from villages randomised to both early and deferred MDA in Myanmar and Vietnam, and from early MDA villages only in Cambodia and Lao PDR. Subscripts in red indicate the number of participants with the *P*. *falciparum PfPailin* genotype [8]—a long haplotype containing *PfKelch13* C580Y, conferring artemisinin resistance, and multiple copies of the *Pfplasmepsin2/3* genotype conferring piperaquine resistance. F/U, follow-up; Lao PDR, Lao People’s Democratic Republic; MDA, mass drug administration; Pf, *P*. *falciparum*.

Before early and deferred MDAs, we identified 269 individuals with *P*. *falciparum* infections, of whom 258 (96%) participated in at least 1 round of DP MDA, i.e., received DP on 3 consecutive days (Fig 5). A follow-up blood specimen was obtained from 221 (86%) participants 1 month after MDA. There were 14 (6%) participants whose infections persisted after treatment (13 in Vietnam and 1 in Cambodia), while the remaining 207 (94%) participants had cleared their infection.

Ten of the 269 (4%) *P*. *falciparum* infections had the *PfPailin* genotype [8] (a long haplotype containing *PfKelch13* C580Y, conferring artemisinin resistance, and multiple copies of the *Pfplasmepsin2/3* genotype conferring piperaquine resistance): 5 of 113 (4%) were in Vietnam and 5 of 8 (63%) were in Cambodia (*p* < 0.001; Fig 5). All but 1 of the 10 participants with *PfPailin* cleared their parasitaemia after receiving at least 1 round of MDA (i.e., clearance rate 90%, 95% CI 56% to 100%). One subclinical *PfPailin* genotype infection in Cambodia persisted after 3 rounds of MDA but by M6 had cleared without further drug treatment.

### Tolerability and adverse events

Following the administration of DP (59,375 individual doses), 121 (0.2%) participants vomited. Of these, 104 vomited shortly after taking the drugs and were offered a repeat dose (Table 3). Data on dizziness and itching were not recorded in Vietnam, but in Cambodia, Lao PDR, and Myanmar, where a total of 26,898 doses of DP were administered, 586 (2.2%) events of dizziness and 12 (0.04%) events of itching were reported following drug administration on day 1. Within 1 month of the MDAs, 1,535 of 8,112 (19%) MDA participants recalled 2,577 AEs, of which 911 (35%) were considered related to the antimalarials; 592 (23%) of the 2,577 AEs were dizziness, 199 (8%) nausea, 96 (4%) vomiting, and 39 (2%) itching, and 1,653 (64%) participants reported a range of other minor complaints. There were no cases of severe haemolysis.

**Table 3:** Adverse events recorded within day 0 to day 3 after MDA using dihydroartemisinin-piperaquine.

Adverse event	Month	Day 0			Day 1			Day 2			Overall		
		Number taking drug	Number of events	Percent of events	Number taking drug	Number of events	Percent of events	Number taking drug	Number of events	Percent of events	Number of doses taken	Number of events	Percent of events
Vomiting	M0	6,866*	51	0.74%	6,769	26	0.38%	6,721	6	0.09%			
	M1	6,583	8	0.12%	6,454	11	0.17%	6,389	4	0.06%	59,375	121	0.20%
	M2	6,583	12	0.18%	6,518	3	0.05%	6,492	0	0.00%			
Dizziness^†^	M0	NA	NA	NA	4,690	176	3.75%	4,680	129	2.76%			
	M1	NA	NA	NA	4,285	87	2.03%	4,247	55	1.30%	26,898	586	2.18%
	M2	NA	NA	NA	4,505	86	1.91%	4,491	53	1.18%			
Itching^†^	M0	NA	NA	NA	4,690	3	0.06%	4,680	6	0.13%			
	M1	NA	NA	NA	4,285	0	0.00%	4,247	1	0.02%	26,898	12	0.04%
	M2	NA	NA	NA	4,505	0	0.00%	4,491	2	0.04%			

*The tolerability data included villages that had MDA but no follow-up (Cambodia and Lao People’s Democratic Republic after M12).

^†^No recorded data from Vietnam for dizziness and itching; data from 1 deferred MDA village from Cambodia included.

M[number], month [number]; MDA, mass drug administration; NA, not applicable.

Within 3 months of the MDA, 6 SAEs leading to death or requiring hospitalisation were reported in villages receiving early MDA (6/4,135 participants; 0.15%), whereas in control villages with deferred MDA, 1 SAE (1/2,596 participants; 0.04%) was reported in the same time span (*p =* 0.187; Table 4). The investigators found none of the SAEs to be related to the administration of study drugs.

**Table 4:** SAEs within 3 months of MDA in villages with early MDA compared to control villages with deferred MDA for the first 12 months.

SAE	Within 3 months			Within 12 months		
	MDA villages (*n =* 4,135)	Control villages (*n =* 2,596*)	Total	MDA villages (*n =* 4,135)	Control villages (*n =* 2,596*)	Total
Death (without diagnosis of underlying disease)	2	0	2	10	5	15
Life-threatening illness	2	1	3	11	3	14
Debilitating disease due to old age	0	0	0	1	0	1
Hospitalisation due to pneumonia	2	0	2	2	0	2
Drowning	0	0	0	0	2	2
Suicide	0	0	0	2	0	2
Gastric cancer	0	0	0	1	0	1
Prolongation of hospitalisation	0	0	0	1	0	1
Total SAEs	6 (0.15%)	1 (0.04%)	7	28 (0.7%)	10 (0.39%)	38

*p* = 0.187 and 0.120 for comparison of number of SAEs between early MDA and control villages within 3 months and within 12 months, respectively.

*No recorded data from Lao People’s Democratic Republic and Cambodia in control villages.

MDA, mass drug administration; SAE, serious adverse event.

## Discussion

This cluster randomised trial demonstrated that in a setting where early diagnosis, effective treatment, and insecticide-treated bednets have already been made available, mass antimalarial drug administration with DP can substantially reduce the transmission of *P*. *falciparum* infections over a 1-year period. This period of protection afforded by the MDA was much longer than the post-treatment prophylactic effect provided by piperaquine (the slowly eliminated and therefore longer acting component of the antimalarial regimen), suggesting that the reduction of the asymptomatic parasite reservoir made a lasting impact on transmission of *P*. *falciparum* infections. An analysis starting 1 month after the last drug dose (i.e., from M3 to M12), after the complete implementation of the intervention and after the prophylactic period, showed a substantial benefit. Twelve months after early DP MDA, the *P*. *falciparum* prevalence had become very low or reached 0 in 4 of 8 intervention villages. However, malaria was also reduced substantially in control villages that did not receive early DP MDA, which was probably related to uninterrupted access to basic malaria control measures in all villages. The intensive community engagement conducted alongside the study activities played a critical role in promoting uptake. Overall, 86% of the target population participated in at least 1 round of the early MDAs, and 81% participated in at least 1 round of the deferred MDAs. In Myanmar and Vietnam, where DP MDA was less effective than in Cambodia and Lao PDR, the proportions of residents participating in all 3 rounds of the MDA was only 57% during the early MDAs and 38% in the deferred MDAs. Completion of the 3-round regimen was significantly associated with a reduction in the risk of becoming infected with *P*. *falciparum* in a multivariable regression model, while completion of a single round was not. Despite widespread artemisinin resistance, we found an overall clearance of 94% of subclinical *P*. *falciparum* infections after 1 or more rounds of MDA. The DP MDA drug regimen with a single low dose of primaquine was safe and remarkably well tolerated. There were no drug-attributable SAEs.

Artemisinin resistance was first reported in western Cambodia in 2008 [27,28], followed 8 years later by the detection of concomitant piperaquine resistance [4,6]. A single co-lineage of parasites (*PfPailin*) has since spread in a broad sweep encompassing northeast Thailand and southern Lao PDR, into southern Vietnam [8]. As a result, the clinical efficacy of DP in symptomatic falciparum malaria in these areas has fallen—often to below 50% [26,58]. Yet in our study, 9 of the 10 individuals with subclinical multidrug-resistant *PfPailin* infections who participated in at least in 1 round of DP MDA cleared their infections. This emphasises the substantial contribution of immunity to drug efficacy in people with asymptomatic malaria and suggests that, at these levels of DP resistance, the drug may still be of value in MDA. However, if malaria transmission in this region continues to increase, it will likely lead to higher levels of resistance, rendering DP progressively less effective. Our findings support the hypothesis that once a large proportion of the subclinical *P*. *falciparum* reservoir has been removed, transmission is reduced or even interrupted completely. This hypothesis is supported by the strong increase in protection with increasing number of MDA rounds in our study, and by recent entomological studies [59].

Where measured directly, the malaria protective effect of long-lasting insecticide-treated bednets in this region has been limited [60]. It has been estimated that in the GMS two-thirds of infective mosquito bites occur outside the home between 5 AM and 9 PM, i.e., where and when bednets are unlikely to be used. This has been confirmed along the Thailand–Myanmar border by the use of serological biomarkers, which show no correlation between bednet use and the human antibody response to malaria vector bites (salivary antigens) or *P*. *falciparum* infections [61,62]. Important local vectors such as *Anopheles maculatus*, *An*. *dirus*, and *An*. *minimus* tend to be exophilic and exophagic [63]. But our study did show an overall significant residual benefit of regular bednet use. This observation could be due to confounding, because irregular bednet use may indicate that the villagers were sleeping unprotected in and around forest edges, which is recognised as a major risk factor for malaria in the region [63–65]. This hypothesis is supported by the observation that only participants aged 12 years and older appeared to be protected by the use of bednets, while bednet use showed no protection in younger children, who are unlikely to participate in forest work (S5 Table).

Our study has a number of limitations. The attrition of the beneficial effect of DP MDA over time in this exploratory study was expected and is related to the study design, where, in each country, a small number of villages located within a malaria endemic area were given DP MDA over 3 months. Residual untreated infections in non-participants, importation of malaria infection from neighbouring untreated villages, or exposure to new infections through travel of villagers to surrounding areas were likely sources of malaria reintroduction [14–16]. Further limitations of the study were the absence of regulatory approval to include a single low dose of primaquine in the drug regimen in Cambodia. In Myanmar, the deferred MDAs took place in 2 villages at M9 instead of M12 because of difficult access in the peak of the rainy season, and the survey at M21 had to be cancelled. Only Myanmar and Vietnam could participate in the surveillance for 1 year after deferred MDAs, due to the delayed start in Cambodia and Lao PDR. The results from the second year are therefore based on a comparison of only 4 versus 4 village populations. Some of the observed higher impact in people who adhered to the complete 3 rounds of MDA compared to people who took part in none or fewer than 3 rounds could be due to the people adhering to the 3 rounds being healthier than the people who did not adhere [66,67]. Furthermore, some study teams did not record reliably AEs/SAEs from control villages because their focus was on the implementation of the MDAs, which may help to explain some of the differences in AE rates between the intervention and control villages.

If MDA is rolled out at the same time in an entire region, the risk of reintroduction of *P*. *falciparum* infections should be much reduced and hence the benefits should be sustained much longer [68]. Timely, accurate diagnosis and the appropriate treatment of residual malaria episodes after completion of MDAs will be essential for the permanent interruption of malaria transmission. This will require the presence of well-supported village health workers who provide several healthcare interventions in order to sustain the motivation for good malaria control as the incidence of malaria illness falls [69]. Further work is still needed to assess the source of *P*. *falciparum* reintroduction after clearing the asymptomatic reservoir, the prevalence thresholds for use of MDA, and the optimum MDA deployment strategies [70,71]. Additional options to achieve and maintain elimination include use of endectocides (i.e., ivermectin) in MDAs to kill vector mosquitoes, and the addition of a malaria vaccine. Even an imperfect vaccine providing a relatively short period of protection could prevent the re-importation of infections during the critical elimination phase [72,73]. Until new antimalarial drugs become available, and while efficacy remains stable at its current level, DP MDA can safely be used in low-transmission zones to accelerate regional elimination of *P*. *falciparum* malaria. Finally, the observation that 3 MDA rounds provide significantly more protection than a single round has direct implications for implementation, suggesting that reducing the 3-round DP MDA regimen to fewer rounds for logistic convenience may be ill advised.

In conclusion, despite imperfect adherence and widespread artemisinin resistance, the DP MDAs in our study were associated with a significant and clinically important long-lasting reduction in *P*. *falciparum* infections. Both the prevalence and incidence of *P*. *falciparum* infections were reduced and became negligible in half of the studied villages. This study, like others, demonstrates the critical importance and challenges of mobilising the target populations to participate in MDAs. To be effective, MDA needs to be part of a comprehensive, well-organised, and well-resourced elimination programme. This requires political will. In the eastern GMS, it is now over 10 years since artemisinin resistance—and the threat it posed to global malaria control and elimination—was recognised. Despite high investment, malaria transmission is increasing, and the antimalarial drugs are failing. The window of opportunity to use DP MDA effectively in the GMS may be closing. Outside the areas where DP resistance has become established, DP MDA could accelerate elimination in malaria hotspots as part of a concerted elimination programme.

## Supporting information

S1 Fig A schematic overview of the study design by study site. (PDF)

S2 Fig CONSORT flow chart including year 2. (PDF)

S3 Fig Prevalence and incidence of *P*. *falciparum* in 8 early MDA and 8 deferred MDA villages by uPCR over a 12-month period followed by an additional 12 months of surveillance in 4 intervention and 4 control villages. (JPG)

S4 Fig Panel *P*. *falciparum* prevalence (%) in 8 intervention (MDA at M0) and 8 control (MDA at M12) villages by uPCR over the 12-month follow-up period. (PDF)

S1 Table Additional information on categories of payment to TME participants/villages across the 5 sites. (PDF)

S2 Table MDA start and end dates. (PDF)

S3 Table Participation in follow-up surveys. (PDF)

S4 Table Multilevel mixed-effects Poisson regression of *P*. *falciparum* infection during follow-up. (PDF)

S5 Table Protection afforded by insecticide-treated bednets by age group. (PDF)

S1 Text Reporting/statistical analytic plan (RAP). (PDF)

## Abbreviations

ACT: artemisinin combination therapy
AE: adverse event
DP: dihydroartemisinin-piperaquine
GMS: Greater Mekong Subregion
ICC: intra-cluster correlation coefficient
IRR: incidence rate ratio
ITT: intention to treat
Lao PDR: Lao People’s Democratic Republic
M[number]: month [number]
MDA: mass drug administration
RDT: rapid diagnostic test
SAE: serious adverse event
TME: targeted malaria elimination
uPCR: ultrasensitive quantitative PCR
