# Supplementary material for: The impact of targeted malaria elimination with mass drug administrations on falciparum malaria in Southeast Asia: A cluster randomised trial
Source: PLoS Med. 2019 Feb 15;16(2):e1002745. doi: 10.1371/journal.pmed.1002745 (PMC6377128; doi:10.1371/journal.pmed.1002745)
Supplement: S1 Fig — (PDF) [file pmed.1002745.s001.pdf]

S1 Figure: A schematic overview of the study design by study site

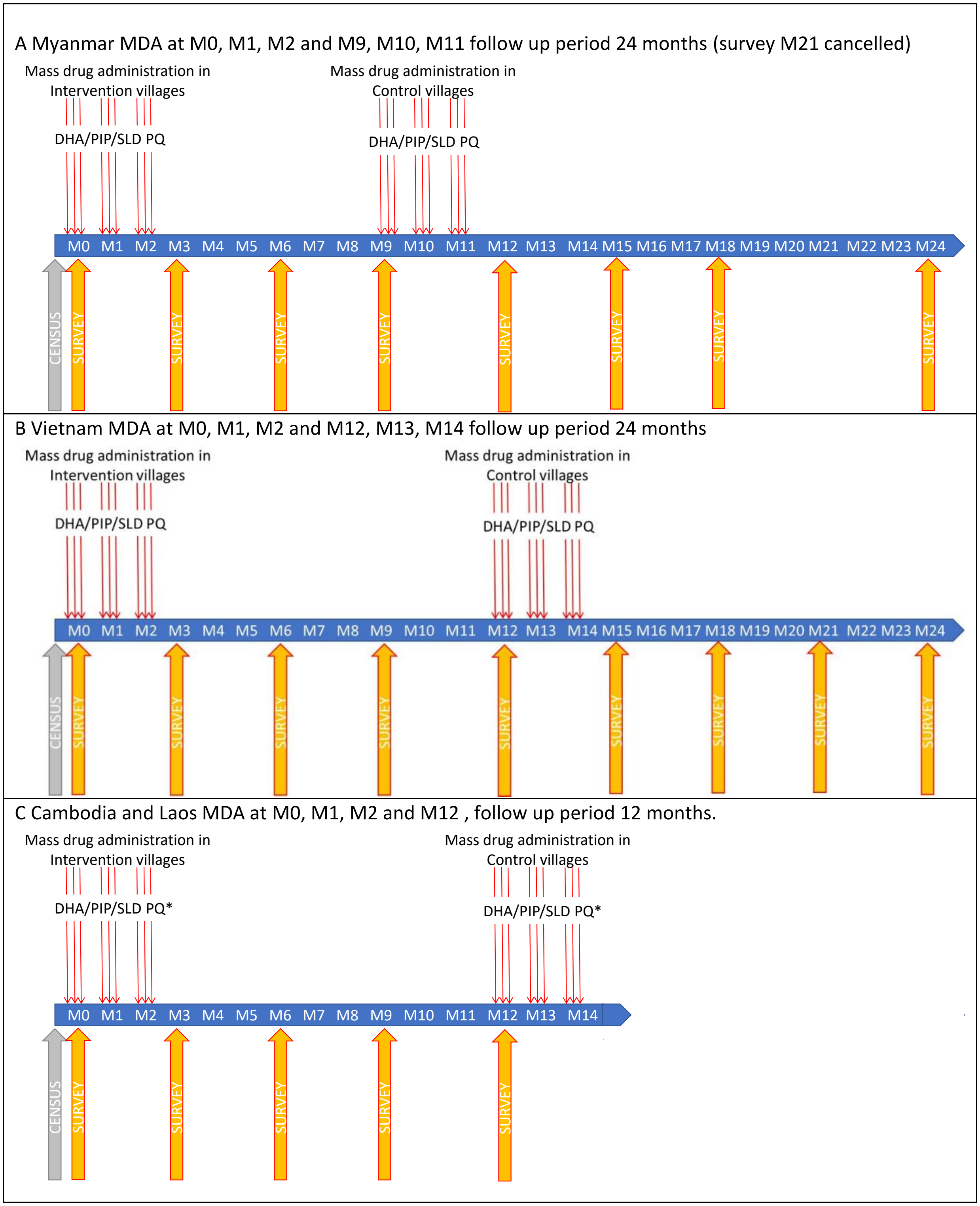

*Single low dose primaquine (SLD PQ) was not administered in Cambodia
