# Supplementary figures and images for: The impact of targeted malaria elimination with mass drug administrations on falciparum malaria in Southeast Asia: A cluster randomised trial
Source: PLoS Med. 2019 Feb 15;16(2):e1002745. doi: 10.1371/journal.pmed.1002745 (PMC6377128; doi:10.1371/journal.pmed.1002745)

S2 Figure: Consort flow chart Year 2

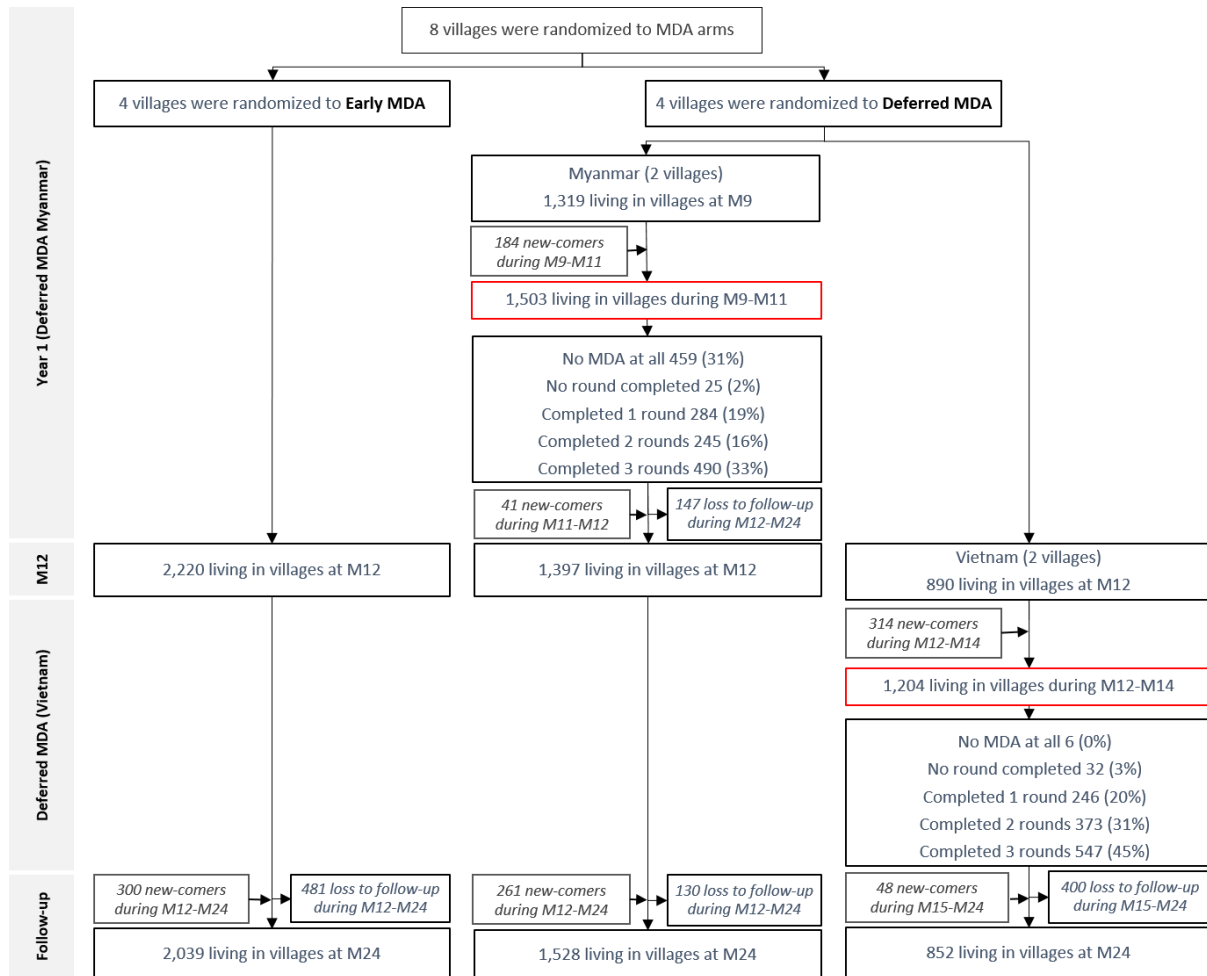

Supplement: S2 Fig — (PDF) [file pmed.1002745.s002.pdf]

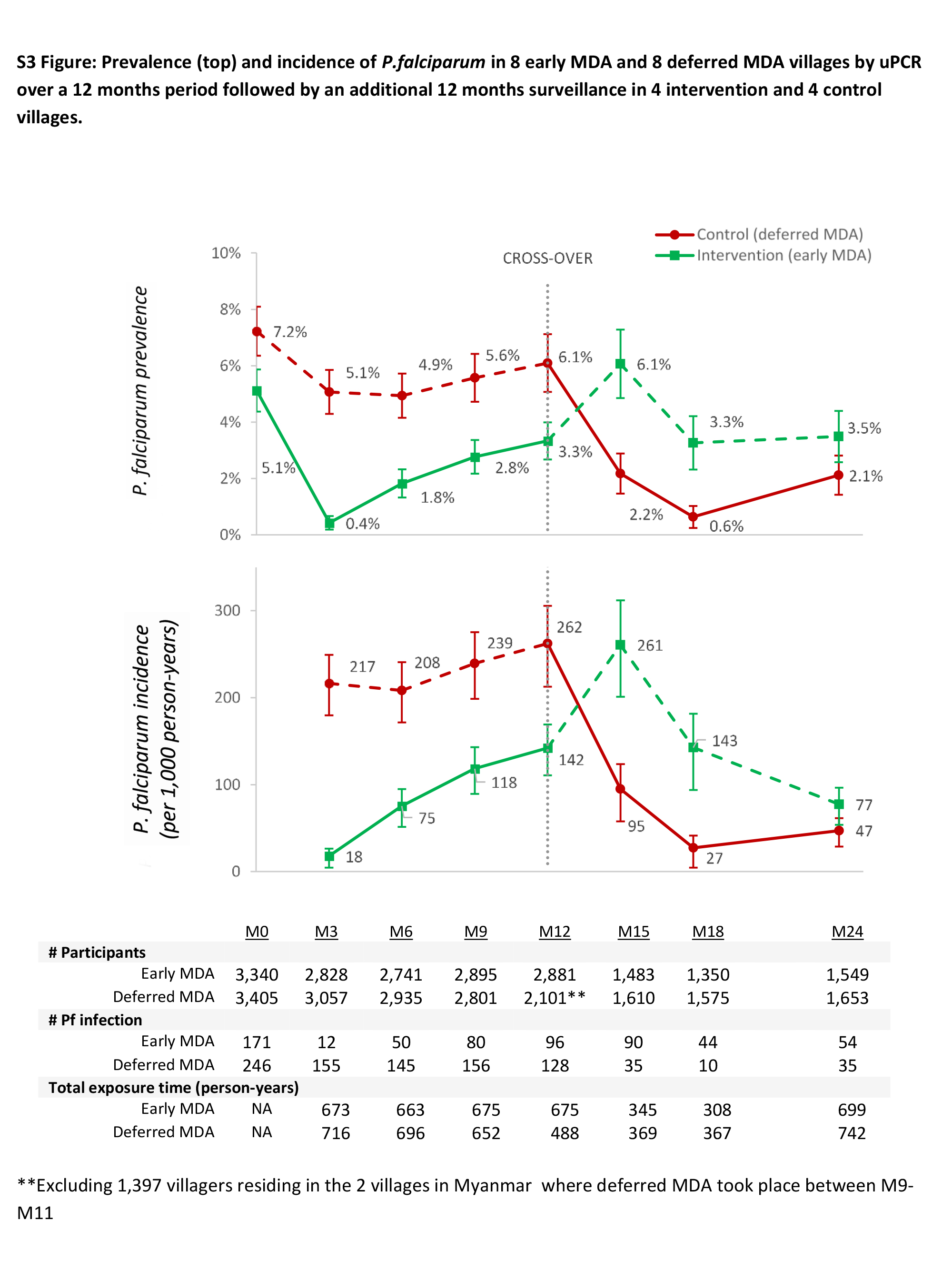

Supplement: S3 Fig — (JPG) [file pmed.1002745.s003.jpg]
