# Supplementary material for: The impact of targeted malaria elimination with mass drug administrations on falciparum malaria in Southeast Asia: A cluster randomised trial
Source: PLoS Med. 2019 Feb 15;16(2):e1002745. doi: 10.1371/journal.pmed.1002745 (PMC6377128; doi:10.1371/journal.pmed.1002745)

S4 Figure: Panel *P.falciparum* prevalence (%) in 8 intervention (MDA at M0) and 8 control villages (MDA at M12) by uPCR over the 12 months follow up period

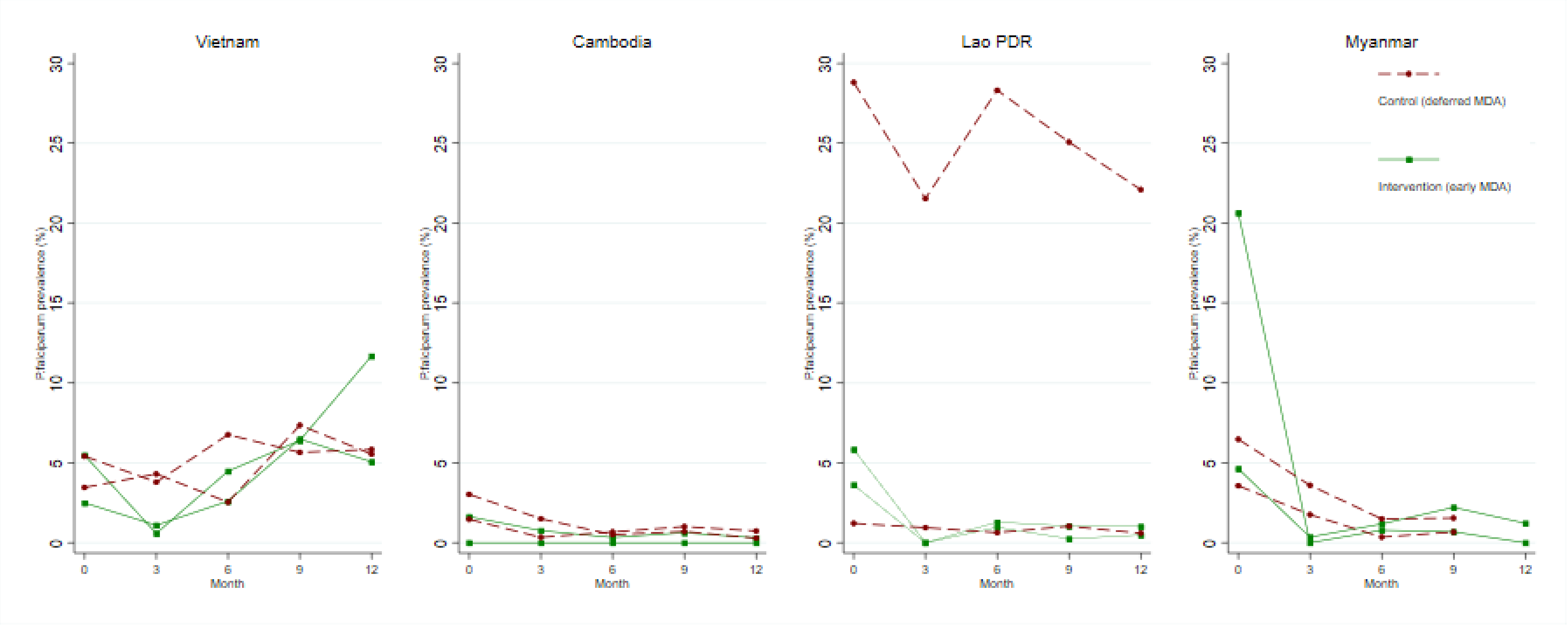

Supplement: S4 Fig — (PDF) [file pmed.1002745.s004.pdf]
