# Supplementary material for: The impact of targeted malaria elimination with mass drug administrations on falciparum malaria in Southeast Asia: A cluster randomised trial
Source: PLoS Med. 2019 Feb 15;16(2):e1002745. doi: 10.1371/journal.pmed.1002745 (PMC6377128; doi:10.1371/journal.pmed.1002745)
Supplement: S1 Table — (PDF) [file pmed.1002745.s005.pdf]

S1 Table: Additional information on categories of payment to TME participants/villages across the 5 sites. **Reimbursement** refers to payment for out of pocket expenses; **compensation** refers to payment or its kinds for time and burden associated with research participants and **incentive** refers to payment or its kinds to motivate participation in TME activities [1].

**Country**		**Lao PDR**	**Cambodia**	**Myanmar**	**Vietnam**
**Pertinent reference**		[2]	[3]	[4]	[5]
**Reimbursement**		Across all TME sites, out of pocket expenses were reimbursed based on their invoices presented. This generally applied expenses related to TME activities for example travel cost for attending meeting conducted in district health centre by health staff and community representatives			
**Compensation* (USD $)**		$ 3.5/day	$ 2.5/day**	None	$ 2.5/day
**Incentives**	**Individual**	T-shirts, cooking utensils, blankets, snacks	Household gift packs, snacks, prizes during quizzes	Snacks	Sweets and rice
	**Village**	Water pumps, free primary health care	Free primary health care	Water storage tanks, free primary health care	Free primary health care

*compensation amount was based on the decisions made with the local researchers
**(only in round 1)

References

1. Gelinas L, Largent EA, Cohen IG, Kornetsky S, Bierer BE, Fernandez Lynch H. A Framework for Ethical Payment to Research Participants. N Engl J Med. 2018;378(8):766-71. Epub 2018/02/22. doi: 10.1056/NEJMsb1710591. PubMed PMID: 29466147.

2. Adhikari B, Phommasone K, Kommarasy P, Soundala X, Souvanthong P, Pongvongsa T, et al. Why do people participate in mass anti-malarial administration? Findings from a qualitative study in Nong District, Savannakhet Province, Lao PDR (Laos). Malar J. 2018;17(1):15. Epub 2018/01/11. doi: 10.1186/s12936-017-2158-4. PubMed PMID: 29316932.

3. Peto TJ, Debackere M, Etienne W, Vernaeve L, Tripura R, Falq G, et al. Community participation during two mass anti-malarial administrations in Cambodia: lessons from a joint workshop. Malaria journal. 2018;17(1):53. Epub 2018/01/29. doi: 10.1186/s12936-018-2202-z. PubMed PMID: 29374462; PubMed Central PMCID: PMCPMC5787251.

4. Kajeechiwa L, Thwin MM, Shee PW, Yee NL, Elvina E, Peapah P, et al. The acceptability of mass administrations of anti-malarial drugs as part of targeted malaria elimination in villages along the Thai-Myanmar border. Malar J. 2016;15(1):494. Epub 2016/09/30. doi: 10.1186/s12936-016-1528-7. PubMed PMID: 27677694; PubMed Central PMCID: PMCPMC5039796.

5. Nguyen TN, Thu PN, Hung NT, Son DH, Tien NT, Van Dung N, et al. Community perceptions of targeted anti-malarial mass drug administrations in two provinces in Vietnam: a quantitative survey. Malar J. 2017;16(1):17. Epub 2017/01/08. doi: 10.1186/s12936-016-1662-2. PubMed PMID: 28061908; PubMed Central PMCID: PMCPMC5216593.
