# Supplementary material for: The impact of targeted malaria elimination with mass drug administrations on falciparum malaria in Southeast Asia: A cluster randomised trial
Source: PLoS Med. 2019 Feb 15;16(2):e1002745. doi: 10.1371/journal.pmed.1002745 (PMC6377128; doi:10.1371/journal.pmed.1002745)
Supplement: S2 Table — (PDF) [file pmed.1002745.s006.pdf]

S2 Table: MDA start and end dates

Early MDA Intervention villages		Vietnam		Cambodia		Lao PDR		Myanmar	
		BK	GIA	B01	B02	LA02	LA03	KNH	TOT
M0 MDA	start	11-Nov-13	08-Jan-14	21-Jul-15	21-Jul-15	01-May-16	21-Apr-16	12-Jun-13	27-May-13
	end	21-Jan-14	20-Mar-14	26-Sep-15	25-Sep-15	27-Jun-16	04-Jul-16	15-Aug-13	05-Aug-13
Deferred MDA Control villages		BB	THA	B03	B04	LA01	LA02	HKT	TPN
M12 MDA	start	16-Dec-14	05-Jan-15	14-Jul-16	22-Jul-16	04-May-17	28-Apr-17	01-Apr-14	28-Jan-14
	end	27-Feb-15	11-Mar-15	19-Sep-16	22-Sep-16	12-Jun-17	01-Jun-17	07-Jun-14	27-Mar-14
