# Supplementary material for: The impact of targeted malaria elimination with mass drug administrations on falciparum malaria in Southeast Asia: A cluster randomised trial
Source: PLoS Med. 2019 Feb 15;16(2):e1002745. doi: 10.1371/journal.pmed.1002745 (PMC6377128; doi:10.1371/journal.pmed.1002745)
Supplement: S3 Table — (PDF) [file pmed.1002745.s007.pdf]

S3 Table: Participation in follow-up surveys

Number of time tested	Frequency	Percentage
1	1,757	20
2	1,144	13
3	1,282	15
4	1,751	20
5	2,815	32
