# Supplementary material for: The impact of targeted malaria elimination with mass drug administrations on falciparum malaria in Southeast Asia: A cluster randomised trial
Source: PLoS Med. 2019 Feb 15;16(2):e1002745. doi: 10.1371/journal.pmed.1002745 (PMC6377128; doi:10.1371/journal.pmed.1002745)
Supplement: S4 Table — (PDF) [file pmed.1002745.s008.pdf]

S4 Table: Multilevel mixed-effects Poisson regression on *P. falciparum* infection during follow-up (month 0 to month 12)

	Univariable model		Multivariable model A: MDA intervention (ITT)		Multivariable model B: MDA Coverage (Dose-related response)	
	IRR (95%CI)	p-value	IRR (95%CI)*	p-value	IRR (95%CI)*	p-value
Model A: Intervention						
MDA village	0.54 (0.21, 1.39)	0.201	0.71 (0.50, 1.01)	0.057		
Non-MDA village	Reference		Reference			
Model B: Coverage						
MDA completed 3 rounds	0.14 (0.05, 0.39)	<0.001			0.30 (0.15, 0.58)	<0.001
MDA completed 2 rounds	0.32 (0.11, 0.90)	0.031			0.60 (0.29, 1.23)	0.160
MDA completed 1 rounds	0.50 (0.17, 1.48)	0.213			0.90 (0.42, 1.90)	0.777
MDA not completed /No MDA	0.56 (0.20, 1.59)	0.275			0.96 (0.47, 1.98)	0.918
Non-MDA	Reference				Reference	
Gender						
Male	1.97 (1.68, 2.31)	<0.001	1.96 (1.66, 2.3)	<0.001	1.64 (1.42, 1.89)	<0.001
Female	Reference		Reference		Reference	
Age (years)	1.00 (1.00, 1.01)	0.042	1.01 (1.00, 1.01)	0.027	1.00 (1.00, 1.01)	0.300
Fever	1.45 (1.19, 1.75)	<0.001	1.49 (1.23, 1.80)	<0.001	1.42 (1.13, 1.79)	0.003
Bednet use	Reference					
Regular	0.98 (0.82, 1.18)	0.859				
Irregular	1.83 (1.34, 2.50)	<0.001				
Never use						
Season	1.10 (1.06, 1.13)	<0.001	1.14 (1.01, 1.28)	0.027	1.07 (0.94, 1.23)	0.312
Wet	Reference		Reference		Reference	
Dry	1.12 (1.10, 1.15)	<0.001	1.12 (1.09, 1.14)	<0.001	1.09 (1.04, 1.14)	0.001
Prevalence of PF infection at baseline in village level						

** Adjusted for all baseline variables except the bednet use because it has about 40% missing data which substantially reduces the sample size for complete case analysis*
