# Supplementary material for: The impact of targeted malaria elimination with mass drug administrations on falciparum malaria in Southeast Asia: A cluster randomised trial
Source: PLoS Med. 2019 Feb 15;16(2):e1002745. doi: 10.1371/journal.pmed.1002745 (PMC6377128; doi:10.1371/journal.pmed.1002745)
Supplement: S5 Table — (PDF) [file pmed.1002745.s009.pdf]

S5 Table: Protection afforded by insecticide treated bednets by age group. Multilevel mixed-effects Poisson regression on *Plasmodium falciparum* infections detected by uPCR during follow-up (month 3 to month 12), in participants age < 12 years versus age ≥12 years.

Characteristics	Age < 12 years		Age ≥ 12 years		Overall	
	IRR (95%CI)	p-value	IRR (95%CI)	p-value	IRR (95%CI)	p-value
Bednet use						
Regular	Reference		Reference		Reference	
Irregular	0.94 (0.63 to 1.41)	0.772	1.54 (1.17 to 2.03)	0.002	1.36 (1.08 to 1.71)	0.009
No use	1.04 (0.39 to 2.75)	0.934	2.23 (1.47 to 3.39)	<0.001	2.02 (1.37 to 2.99)	<0.001
