# Supplementary material for: The impact of targeted malaria elimination with mass drug administrations on falciparum malaria in Southeast Asia: A cluster randomised trial
Source: PLoS Med. 2019 Feb 15;16(2):e1002745. doi: 10.1371/journal.pmed.1002745 (PMC6377128; doi:10.1371/journal.pmed.1002745)
Supplement: S1 Text — (PDF) [file pmed.1002745.s010.pdf]

Analysis of TME studies: Analytical Plan

Cluster- Randomised Controlled Trial of Mass Drug Administrations
for the elimination of Pf malaria

TABLE OF CONTENTS

OVERVIEW 3
1.1 Corresponding protocols: 3
1.2 Intervention 3
1.3 Indication: 3
1.4 Sponsor: 3
SUMMARY 5
OVERVIEW OF ANALYSIS PLAN 9
Denominators 13
Endpoints 19
Definitions 20
1. Definition of principal outcome events 20
2. Other a priori definitions 21
IMPACT ANALYSIS 23
CANDIATE VARIABLES FOR INCLUSION AS COVARIATES 25

OVERVIEW

1.1 Corresponding protocols:

- Targeted Chemo-elimination of malaria (TCE), OXTREC 1017-13, version 1.3, 22 January 2015 (Targeted chemo-elimination (TCE) to eradicate malaria in areas of suspected or proven artemisinin resistance in Southeast Asia and South Asia)
- Defining the micro-epidemiology and elimination strategy of falciparum malaria in areas of artemisinin resistance in Pailin, Western Cambodia, Version 1.1 25 April 2013 OXTREC 1015-13
- Targeted mass treatment (TMT) in areas of suspected or proven artemisinin resistance in Kayin State, Myanmar

1.2 Intervention

Presumptive antimalarial therapy of entire villages

Study drugs: dihydroartemisinin/piperaquine + single, low-dose primaquine

1.3 Indication:

Malaria elimination

1.4 Sponsor:

Oxford University via Mahidol-Oxford Research Unit (MORU), Bangkok, Thailand

1.5 Study phase:

Phase 3b Effectiveness evaluation (multi-site)

1.6 Principal Investigators:

Nick White

Arjen Dondorp

1.7 Co-Investigators:

Francois Nosten, Shoklo Malaria Research Unit (SMRU)

Prof. Hien, Oxford University Clinical Research Unit (OUCRU)

Mayfong Mayxay, Lao Oxford Mahosot Hospital Wellcome Trust Research Unit (LOMWRU)

Frank Smithuis, Myanmar Oxford Clinical Research Unit (MOCRU)

SUMMARY

This document describes the specific procedures to be followed in the reporting and analysis of the above-mentioned trial and the format of data output.

2. Study Synopsis

TME study sites are Thai-Myanmar border areas (SMRU), Vietnam, Cambodia, Laos, and South Karen State, Eastern Myanmar (SKS/MAM). A more detailed description of study sites is provided below.

Description of the Study sites

Vietnam

- Four villages in two communes have been selected based on relatively high parasite prevalence and a positive environment for the project:
    - BK village (intervention) BB (control) Dak O commune; Binh Phuoc Province
    - GIA village (intervention) THA (control) Phuoc Ha commune, Ninh Thuan Province

Thai- Myanmar Border-areas (SMRU)

- Four villages selected based on relatively high parasite prevalence and a positive environment for the project:
    - KNH and TOT (intervention)
    - HKT and TPN (control)

Cambodia: Battambang province

- Four villages

Laos: Nong district, Savannakhet province

- LA01= Oi Tan Tip
- LA02=Phounmakmee
- LA03=Thate
- LA04=Xuang Tai

Myanmar: Kyaingseikgyi, South Karen State, Eastern Myanmar (SKS/MAM).

- 18 villages (10 villages for drug administrations and 8 villages controls)

Trial methodology:

- **Village selection:** A pre-screening survey is conducted in around 20 villages. Approximately 50 volunteer residents per village are tested by uPCR for parasitaemia. 4 villages are selected based on parasite prevalence, enthusiasm of villagers to participate, accessibility, etc.
- **Restricted randomisation:** Two villages are matched based on geography, accessibility, and parasite prevalence. One village in each pair is selected for intervention using a computer-generated randomisation plan. The other villages serve as controls.
- **The follow-up period** is 24 months in the villages in SMRU and Vietnam and 12 months in Cambodia, Laos, and SKS/MAM.
- **Cross-over** at 12 months in Vietnam and at 9 months in SMRU at which time the control villages receive the intervention and the villages initially randomised to the intervention serve as “controls”. The second follow-up period is 12 months after cross-over in Vietnam and SMRU. Total follow-up is for 2 years in Vietnam and SMRU.
- **Surveys:** All villagers are tested every 3 months using uPCR.
- **A cohort** consisting of all participants found to be parasitaemic during the first survey are tested monthly for the study period in Vietnam and Laos (not SKS/MAM, Cambodia and SMRU).

Study drugs / intervention

- 3 rounds of 3 days DHA/piperaquine are given in monthly intervals (Month 0, Month 1, Month 2)
  - One DP tablet contains 40 mg of dihydroartemisinin and 320 mg piperaquine. A weight-based regimen containing a total dose of approximately 7 mg/kg dihydroartemisinin and 55 mg/kg piperaquine phosphate will be used.

- Combined with 3 rounds of a single, low dose primaquine
  - PQ 0.25 mg/kg single dose will be administered on day 1 or day 3 of the 3 days DP regimen in all study sites except in Cambodia.

Table 1

Rounds	Doses	Abbreviation
1st round (month 0)	1st dose<br>2nd dose<br>3rd dose	R1-D1<br>R1-D2<br>R1-D3
2nd round (month 1)	1st dose<br>2nd dose<br>3rd dose	R2-D1<br>R2-D2<br>R2-D3
3rd round (month 2)	1st dose<br>2nd dose<br>3rd dose	R3-D1<br>R3-D2<br>R3-D3

OVERVIEW OF ANALYSIS PLAN

The objective of this multi-centre study was the elimination of Pf malaria. The primary outcome will be calculated as an incidence for Pf and mixed Pf+Pv infections during the first follow-up period using the time contributed by each villager as the denominator. Infections will include participants with parasites that were detected by any method (uPCR, RDT and microscopy) at any time during the study period (i.e., positive infections recorded by VMWs will be included when these data are available). Results in the treatment villages will be stratified by the number of MDA rounds received to assess effectiveness. Other effectiveness measures that will be examined include the number of times infected with Pf in the first follow-up period (12 months for most study sites) and the time to first Pf infection. These outcomes will provide insight into how long one might expect MDA to protect against re-infection.

Estimates of Pf incidence will be presented for each village, by study site and pooled across all sites and villages. Incidences will be presented graphically using forest plots to assist in the determination of the most appropriate analytical approach.

It is anticipated that results will be reported as Incidence Rate Ratios with 95% confidence intervals (CI). A mixed effects Poisson model will be used to model the incidence of Pf infection. Data should be arranged such that the individual level outcome (Pf positivity 0/1) is available at each time point. Each time point will also contain the exposure variable (person-time of observation in DAYS) (see example, Table 1). Univariate analyses will be performed to obtain crude (unadjusted) estimates of the relationship between the incidence of Pf and each of the explanatory variables (covariates) of interest. The covariates to be included is based on consensus among the investigators (see table XX).

This will be followed by a multivariable (multivariate) analysis to obtain the adjusted estimates of the incident rate ratios. All variables significant in the univariate analysis will be candidates for multivariable analysis. In addition, any covariates which are thought to be a priori confounders, e.g., age and sex, will be included in the multivariable analysis. In the case where the mixed

effects models do not converge or are not able to provide stable estimates, GEE (population averaged) models will be considered as an alternative, but not before specific consultation with the study statistician. A p-value of 0.05 will be considered significant.

A subgroup analysis of the cohort found to be parasitaemic during the first survey in Vietnam and Laos will also be conducted following the analytic approach used for the work in Pailin by Tripura and co-workers.[1]

Table 1 Data presentation in long format.

Site	village	treat (1=MDA 0=Control)	ID	Time (survey month)	Pf (0/1)	Cum. person-time (days)	covariate 1 e.g. age	cov e.g.
1	1	1	1	0	0	0	10	
1	1	1	1	3	0	91.25	15	
1	1	1	1	6	1	228.13	62	
1	1	1	1	12	1	273.76	11	
1	1	1	2	0	0	0	46	
1	1	1	2	3	0	91.25	33	
1	1	1	2	6	0	182.5	38	
1	1	1	2	9	1	273.75	20	
2	2	0	1001	0	1	0	21	
2	2	0	1001	3	1	91.25	17	
2	2	0	1002	0	1	0	8	
2	2	0	1002	3	0	91.25	7	
2	2	0	1002	6	1	182.5	73	

The following commands may provide a starting point in the analysis for STATA users:

```
mepoisson pf_yesno treat age sex other_covariates,
exposure(exposure_time) irr
```

OR

```
mepoisson pf_yesno treat age sex other_covariates,
exposure(exposure_time) irr ||  village:
```

[1] Persistent *Plasmodium falciparum* and *P. vivax* infections in a western Cambodian population: implications for prevention, treatment and elimination strategies Rupam Tripura , Thomas J Peto, Jeremy Chalk, Sue J Lee, 2, Pasathorn Sirithiranont, Chea Nguon, Mehul Dhorda, Lorenz von Seidlein, Richard J Maude, Nicholas PJ Day, Mallika Imwong, Nicholas J White, Arjen M Dondorp MJ in press

Similar programs may also be developed in R for R-users while allowing for the same modelling approach across sites.

Example Tables for presentation of results

These example tables should be presented for each village, site and with pooled estimates across all sites.

Table. Baseline and demographic summary for Site.

**Variables**	**MDA** (n= 2 villages)	**Control** (n= 2 villages)	**Overall** (n= 4 villages)
Pv prevalence, n (%)			
Pf prevalence n (%)			
Age in years: median (range)			
Sex: Female n (%)			
Etc.			

Table. Univariate and multivariate analysis for Village.

**Rank**	**Co-variate**	**Unadjusted IRR**	**95% CI**	**P-value**	**Adjusted IRR**	**95% CI**	**P-value**
1	Intervention coverage: percent of residents completed MDA (9 doses)*						
2	Newcomer/arrival rate						
3	Baseline Pf Prevalence (village)						
(4	Province Pf incidence)						
5	Pv incidence / prevalence						
6	Pv prevalence						
7	Bednet coverage						
8	Sex						
9	Age						
10	Departure rate						
11	Malaria post visitors rate						
12	Prevalence of Pf						

	parasitaemia<br><br>in MP visitors and newcomers						
13	Pregnant women/fixed non-participants						
14	Season wet/dry						

** Applies to intervention villages only*

Denominators

Two denominator variables are considered: a) the people and b) the time they contribute. An agreed denominator from these two options will be used across sites to allow for consistency of reporting of the results. The choice will be determined by the availability of the data for the chosen option across the sites and on the practicalities of handling participants with partial observed times during the study period.

1. **Census**

Two censuses, one at the beginning and a second at the end of the study period are conducted in each study village. Each census captured the *de jure* population (defined as persons who stated their residence in the study area was their regular residence).

Active Demographic Surveillance Updates

Exhaustive surveys are conducted in the entire village population at 3 monthly intervals (M0, M3, M6, M9, and M12 for all villages and in addition M15, M18, M21, M24 in Vietnamese study villages and M15, M18 and M24 in SMRU villages. Demographic information (present/absent) is collected during each survey.

Definitions for Derived Demographic Events

Permanent resident: is present for more than 50% of the exhaustive surveys.

Temporary resident: is present for less than 50% of the exhaustive surveys.

Visitor: no evidence for a visit for more than single day, e.g. visit to malaria post or present only at one survey

In-migrants are treated as temporary or permanent residents based on the time they spend in the village (e.g. students).

Newborns: are treated as in-migrants in the analysis

Moved away for good/final (MAF): includes permanent out-migrants and deaths

2. **Populations of interest**

Population for incidence calculation:

Primary analysis

1. includes all participants for whom an amount of time present during the study period can be calculated (including VMW data) during the first follow-up period. This the most inclusive approach and most relevant for the policymakers having to decide whether to implement the intervention.

Secondary analyses

1. Only includes people who started at M0. This will show the direct effect of the intervention and the duration of protection.

2. Permanent + temporary residents only (i.e., exclude visitors), per jordi's approach. This is the best documented population and will provide the most robust data.

Total population: includes temporary and permanent residents, as well as newcomers (visitors, in-migrants and newborns) and VMW visit data. Used to assess the mobility of a population. flow chart useful here?

Total exposed population = permanent residents + temporary residents + visitors., i.e., anyone in the village on the index day who was seen and recorded. Used for coverage estimates using census data (total exposed population/(census – MAF)).

Example showing "flow" of in and out movement from villages during surveys

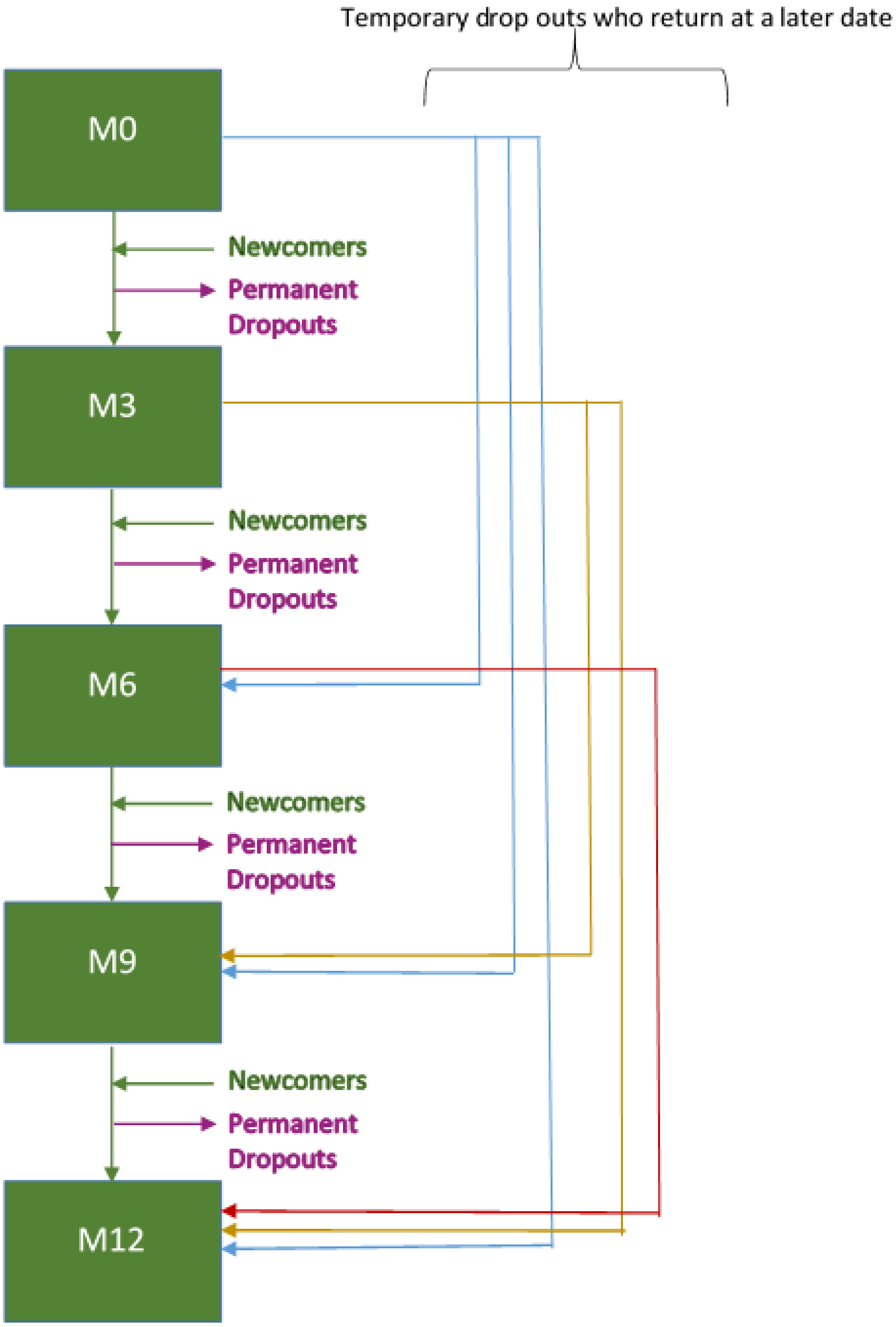

Example flow chart showing coverage from Total Exposed Population

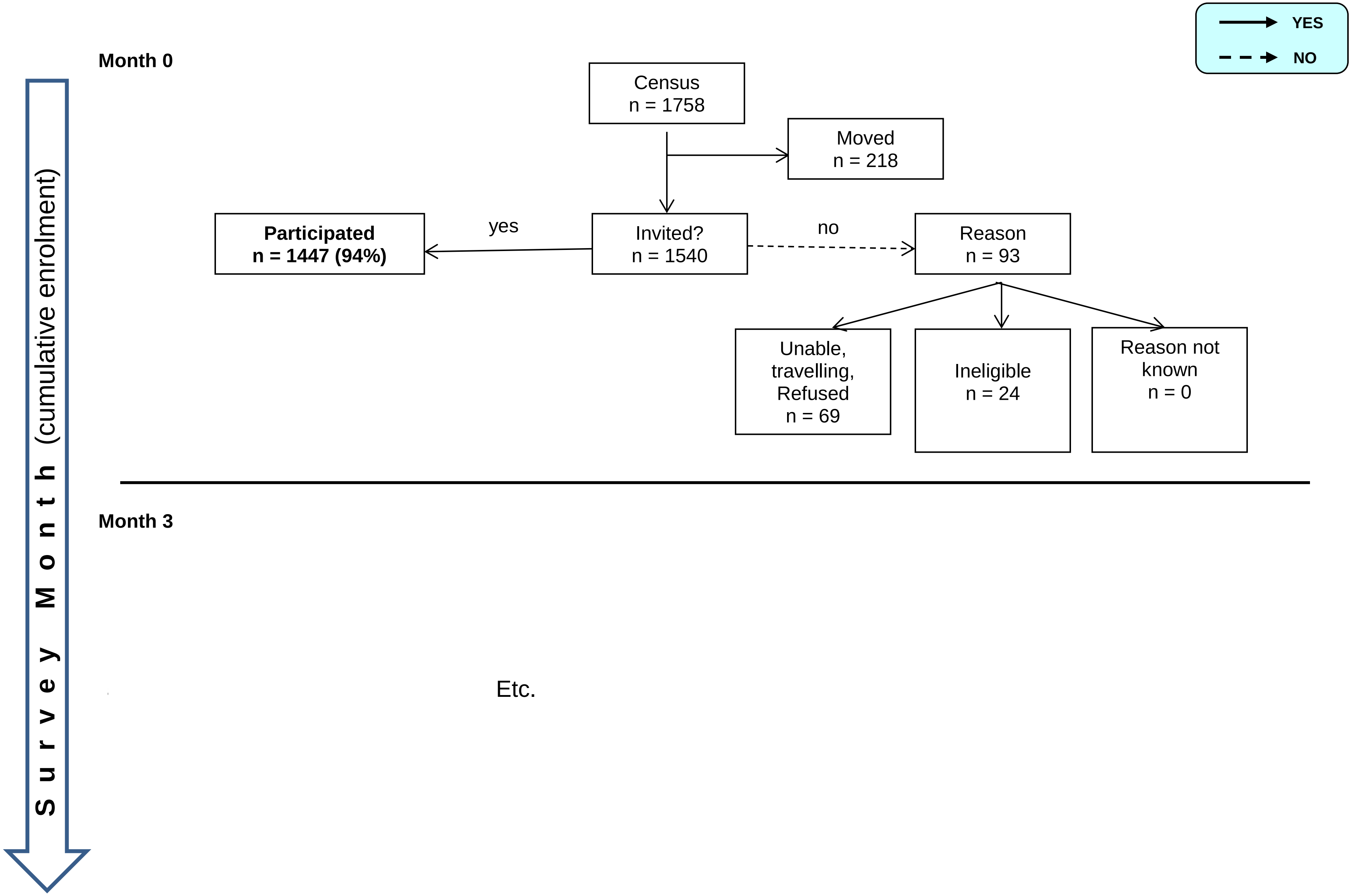

Example table showing coverage from Total Exposed Population (Pailin)

Survey	cumulative enrolment/ census	moved away >1 month	Invited *	Travel**, Unable Refused	Reason not known	In-eligible	Participated †	Coverage††
M0	1758	218	1540	69	0	24	1447	94%
M3	1992	469	1523	88	4	46	1385	91%
M6	2125	573	1552	263	0	44	1245	80%
M9	2230	845	1385	100	4	36	1245	90%
M11	2330	829	1501	193	0	41	1266	84%

* Includes all villagers who were not away from the village for more than 1 month; **short travel away for <1 month;
† provided a blood sample; † † coverage = participated/invited

Time

The time contributed by each resident in the village is assessed for the primary outcome. The time will be calculated in days and reported in person-weeks. Each month is considered to have approximately 30.4 days, therefore month 0 to month 3 would constitute approximately 91.25 days and a villager who is present for all 5 surveys will have a person-time of 365 days (52 weeks). The exact number of days can be calculated from the date of admission to the study to the date of next follow-up, etc.

A resident who has been present for each demographic event during the first follow-up period contributes 12 months (52 weeks) in Cam/Laos/ SKS-MYN and Vietnam and 9 months (39 weeks) in SMRU. Only the first follow-up period will be included in the combined analysis. If the departure and return dates are known the precise dates are used to *censor* the period of absence. If a resident misses a survey due to absence (i.e. not due to refusal to participate) and the departure and return dates are unknown we estimate that the participant has been absent from the midpoint between the last survey in which the participant participated and the first survey when the resident was absent. The time between these time points is *censored*, i.e., does not contribute to the denominator. Similarly when the resident returns we estimate that the resident returns at the midpoint between the last survey when the resident was absent and the first survey when the resident was again present. The same assumptions apply to newcomers, including newborns, for whom the date of arrival is unknown and departures, including deaths, for whom the exact date of death is unknown.

Figure 1: Overview surveys in SMRU and Vietnam

	-1	0	1	2	3	4	5	6	7	8	9	10	11	12	13	14	15	16	17	18	19	20	21	22	23	24	25
census 1																											
close out census 2																											
surveys																											

Figure 2: example time assumptions for a resident who misses one survey (M6). Light blue is the time assumed absent

surveys	M3												M6												M9
weeks	1	2	3	4	5	6	7	8	9	10	11	12	1	2	3	4	5	6	7	8	9	10	11	12	

Analogously for a resident who participates for the first time in the M6 survey and all subsequent surveys we assume/estimate that the resident arrived at the midpoint between the previous (M3) and the present M6 survey. The same assumptions are made for a departing resident – we estimate the resident leaves midpoint between the last survey at which the resident was present and the next survey when the resident was absent. The same time contributions are estimated for residents who miss multiple surveys.

Note: precise dates replace estimates whenever available.

Endpoints

Primary endpoint

- Originally stated as (in registration documents) "**Prevalence** of falciparum malaria in each village measured by qPCR, 12 months after the first administration of TCE." This endpoint selection has been complicated by the fact that in SMRU crossover took place at Month 9. Since the control villages were treated at M9 it is no longer possible to compare the prevalence of treated and untreated villages at M12. Prevalences at M9 or M12 may still be reported by site but as secondary endpoints because of the differences in crossover times as explained above.
- The revised primary endpoint is the **incidence** (person-months) of *P.falciparum* and mixed *P.falciparum* + *P.vivax* parasitaemia at the end of the first follow-up period.

Secondary endpoints will be:

- **Safety / tolerability** evaluated by questionnaires filled out by participants or care givers:
    - Adverse events (AE) and severe adverse events (SAE) within 1 hour of drug administration
    - AE/SAE within 7 days of drug administration

- **Acceptability** will be:
    - Quantified as coverage in the treatment villages (=n/d; please see also definitions of numerator and denominator below).
        - Numerator (n): Number (%) of eligible residents who completed all 3 , 2, 1 , or no round of MDA (permanent residents + temporary residents + visitors, i.e., total exposed population in village)
        - Denominator (d): all village residents (temporary or permanent) who were present for at least one round of treatment
          Note: People who can be included in the numerator have also have to be included in the denominator.

    - Acceptability will also be evaluated by questionnaires filled out by participants or care givers. In some sites acceptability will be assessed in qualitative studies.

- Effect on **gametocyte carriage** (M0, M3, M6, M9, M12, M15, M18, M21, M24) where microscopy results are available. Molecular methods to detect gametocyte specific RNA are in development but may not be ready for the purposes of analysis described here.

- **Molecular analysis** to determine the frequency of specific parasite genotypes, markers of resistance and parasite population genetic structure. Compare and contrast parasite populations before / after the intervention to explore the proportion of re-imported *vs.* persistent parasites.
- **Herd protection** based on the risk for parasite prevalence in geographically defined areas/strata with various a spectrum of coverage/participation in the MDA. The hypothesis is that individuals living in strata with very high coverage are at a much lower risk than people living in areas with minimal coverage.
- The **percentage of people who remain free of Pf** from the moment the intervention is started.
- **Initial prevalence and prevalence at the end of the each follow-up period**.
- **Prevalence for the subgroup of newcomers** (visitors and in-migrants)
- **Logistics, costs and feasibility**
    - Resources, including personnel, needed for drug administrations are recorded and analysed.

Definitions

1. Definition of principal outcome events

- **Parasitaemia detection of parasites in blood specimens**
    1. *P. falciparum* (Pf)
    2. *P. vivax* (Pv)
    3. Mixed infection Pv +Pf
    4. Species not identified (P. spp) – the Pf/Pv ratio of P.spp is assumed to be similar to the Pv/Pf ratio detected by uPCR.

    Parasitaemia can be detected using:
    - High volume ultra-sensitive quantitative polymerase chain reaction (uPCR)
    - Rapid diagnostic tests (RDT)
    - Microscopy

- **Malaria = Parasitaemia + fever** (= temp >=37.5 degrees celcius regardless of method of measurement) or history of fever
    1. *Falciparum* malaria
    2. *Vivax* malaria

3. mixed infection malaria
4. unspecified malaria

2. *Other a priori definitions*

Demographic groups - definitions provided above (see p. 13-14)
Cluster / Village: The study is a cluster randomized trial. The unit of the randomization is a *cluster*. A cluster is a collection of geographically contiguous households in the form of a village.

Month Zero (M0): The *zero time* is the beginning of the study period defined separately for intervention and control villages.

Zero time: For intervention villages, the *zero time* is the first day of the first drug administration; it is the zero time date of the matched intervention village.

Month 3 (M3) This is the first exhaustive survey after M0. Usually this takes place three months after the first day of the M0 survey.

Exhaustive survey: an Active Demographic Surveillance update which includes all village residents. The surveys include the collection of blood samples and interviews. The reason for absence in the survey is recorded.

Age- and residence- eligibility for the trial: Persons present at zero time, as reflected in the pre-drug administration census, and aged >=6 months at the *zero time* were eligible to receive the drug.

Medical exclusion criteria for the trial: Individuals with one or more medical contraindications to either of the drugs at *zero* time were excluded. Pregnant women did not receive primaquine. Residents with a history of allergy or known contraindication to artemisinins, piperaquine or PQ were excluded.

Non participants: includes absentees, refusers and exclusions

Absentees: Absentees are people known to reside in the village during drug administration but failed to be contacted by teams recruiting subjects for drug administration. These people are either in the pre-drug administration census or are noted retrospectively in the **Active Demographic Surveillance Updates** (exhaustive

surveys – see also below) to have been residing in the study area at the time of drug administration.

Refusers: The trial requires signed informed consent for participation. Residents and children present in the village but whose parents refuse to sign informed consent for participation are considered refusers.

Exclusions: children <= 6 mos and villagers with a contraindications to artemisinins, piperaquine or primiquine. Self-reported preganant or breastfeeding women.

Censuses:

- **Initial census** is carried out to collect the household information before M0. These censuses capture the *de jure* population (defined as persons who stated that their residence in the study area was their customary residence) and recorded the demographic events of the population in the study area.
- **Active Demographic Surveillance Updates** provide information on the population in the village on the day of each exhaustive survey (M0, 3, 6, 9, 12) and 15, 18, 21, and 24 in Vietnam and 15, 18, and 24 in SMRU. The information collected during the surveys is the *de facto* census of people actually in the village at the time of the survey.
- The **final census** was termed the **close-out census** (M12 in all sites except SMRU and Vietnam where it is M24).

Randomized population: Residents who were age-eligible and known to be present at *zero time*, either by being in the pre-drug administration census or by being retrospectively identified as having been present at *zero time* in the post-drug administration censuses/surveys, were considered randomized according to the assignment of the cluster of residence at *zero time*.
Age- and residence- eligible population for surveillance: People present in the pre-drug administration census and aged >= 6 m.o. at the time of onset of the disease during the follow up period were eligible to be included in the analysis.

Loss to follow up: Those who moved out from the study area during the follow up period (see also Moved away for good/final (MAF), p. 13 above).

Pre-drug administration period: Pre-drug administration period is the period before the *zero time*.

Study follow-up period: Follow-up begins at *zero time*. The duration of follow-up *for the primary endpoint* was 365 days after *zero time* for Myanmar, Cambodia, Vietnam and Laos and 9 months after zero time for SMRU. In Vietnam and SMRU, there was a second follow-up period of 12 months after assessment of the primary endpoint.

Post-follow-up period: The time after the completion of follow-up period is defined as post-follow-up period. Onset of events occurring in this period will not be considered for analyses.

Definitions of fever: a temperature greater than or equal to 37.5 (however measured) or a reported history of fever.

- **Objective Fever:** An axillary temperature ≥37.5°C.
- **Subjective Fever:** Historical recall of having fever

IMPACT ANALYSIS

The analytic approach is analogous to a ***per protocol*** analysis.

1. **Primary outcome**

Onset of follow-up for counting outcome events: parasitaemia episodes will only be included in the analysis if they have onsets between the first day of drug administrations and the last day of the first follow-up period.

Duration of the first follow up period: 12 months for Laos, Cambodia, Myanmar and Vietnam, 9 months for SMRU control villages

Total Impact is estimated by comparing the incidence of the outcome (Pf or mixed Pf +Pv infections) among the total exposed population in the intervention villages and the incidence of the outcome in control villages. Only residents are included in the analysis. Visitors who have no realistic opportunity to contribute to the transmission of malaria are not included. Whether residents satisfied the inclusion/exclusion criteria, followed the protocol, and received the complete, correct doses is irrelevant for the inclusion in the denominator.

Numerator events for analysis of total impact: Numerator events will be the principal outcome events (Pf or mixed Pf +Pv infections). The analysis will be repeated for each of the principal outcome events Pv, P. spp and for symptomatic malaria.

Denominator for analysis: all residents who could have realistically contributed to the transmission of malaria (total exposed population).

2. **Subgroup analyses** (see also populations of interest p13)

The analysis will be repeated for:

1. participants who took the complete course of 9 doses MDA,
2. participants who completed 2 rounds,
3. participants who completed one round
4. residents who didn't complete a single round.
5. "newcomers", i.e., those who were surveyed for the first time after zero time.

CANDIATE VARIABLES FOR INCLUSION AS COVARIATES

- To be used in final multivariable models

Candidate zero time variables for inclusion as covariates in multivariable models are listed below. Tables will be prepared comparing the MDA and control villages at zero time with respect to cluster level and individual level characteristics.

Candidate variables for exploration and their categories:

Rank	Co-variate	category
1	Intervention coverage: percent of residents completed MDA (9 doses)	% village
2	Newcomer/arrival rate	Number/people/year
3	Baseline Pf Prevalence (village)	Cases/ people
4	Province Pf incidence	Case/ people/ year
5	Pv incidence / prevalence	Case/ people/ year
6	Pv prevalence	Cases/ people
7	Bednet coverage	% village
8	Sex	0: female<br>1: male
9	Age	In years (continuous)
10	Departure rate	Number/people/year
11	Malaria post visitors rate	Number/people/year
12	Prevalence of Pf parasitaemia in MP visitors and newcomers	Cases/people
13	Pregnant ladies/fixed non-participants	Cases/people
14	Season wet/dry	0: wet<br>1: dry
